# Long‐Term Expandable Periosteal Organoids Reconstruct a Functional Regenerative Niche That Support Hematopoietic Stem Cell Maintenance and Bone Repair

**DOI:** 10.1002/advs.78380

**Published:** 2026-10-22

**Authors:** Jia Qing, Lanxin Gu, Lipeng Fu, Xin Gao, Zechuan Li, Hui Zhang, Wenpeng Song, Huixin Lv, Yongsheng Zhou, Ping Zhang

**Affiliations:** ^1^ Department of Prosthodontics Peking University School and Hospital of Stomatology & National Center of Stomatology & National Clinical Research Center for Oral Diseases & National Engineering Research Center of Oral Biomaterials and Digital Medical Devices Beijing China; ^2^ Beijing Key Laboratory for Intelligent Biomanufacturing and Regeneration of Craniofacial Tissues & NHC Key Laboratory of Digital Stomatology Beijing China; ^3^ Institute of Advanced Clinical Medicine Peking University Beijing China; ^4^ Chongqing Key Laboratory for Oral Diseases and Biomedical Sciences Chongqing Municipal Key Laboratory for Oral Biomedical Engineering of Higher Education Stomatological Hospital of Chongqing Medical University Chongqing China

**Keywords:** bone repair, hematopoietic stem cell niche, long‐term expansion, periosteal organoid, skeletal regeneration

## Abstract

The periosteum plays a central role in skeletal regeneration by serving as a reservoir of skeletal stem cells and a dynamic regenerative niche. However, in vitro models that faithfully preserve its cellular complexity, long‐term stability, and functional capacity remain limited, restricting mechanistic studies and translational applications. Here, we establish a long‐term expandable periosteal organoid system that can be stably propagated for over 20 passages while preserving cellular heterogeneity, multilineage differentiation potential, and genomic integrity. Single‐cell transcriptomic analysis reveals sustained maintenance of core periosteal lineages and regenerative signaling programs over extended culture. Functionally, these organoids reconstruct a niche‐like microenvironment that supports hematopoietic stem cell (HSC) maintenance, preserves stem cell fitness, and attenuates aging‐associated functional decline under prolonged ex vivo conditions. Upon transplantation, periosteal organoids robustly engraft and actively participate in tissue repair, promoting vascularized bone formation and reconstruction of marrow‐like structures across multiple defect models. Mechanistically, we identify ZFP575 as a 3D niche‐dependent regulator required for maintaining in vivo osteogenic competence. Together, our findings establish periosteal organoids as a physiologically relevant and functionally competent regenerative platform, providing a versatile system for modeling skeletal repair and stem cell‐niche interactions.

## Graphical abstract

A robust periosteal organoid system enables long‐term expansion while preserving native cellular heterogeneity. These organoids recapitulate functional niche environments, support hematopoietic stem cell maintenance, and attenuate aging‐associated decline ex vivo. Even after extended passaging, they drive vascularized bone formation with marrow reconstruction upon transplantation, with the Zfp575 transcription factor governing osteogenic competence and engraftment outcomes.

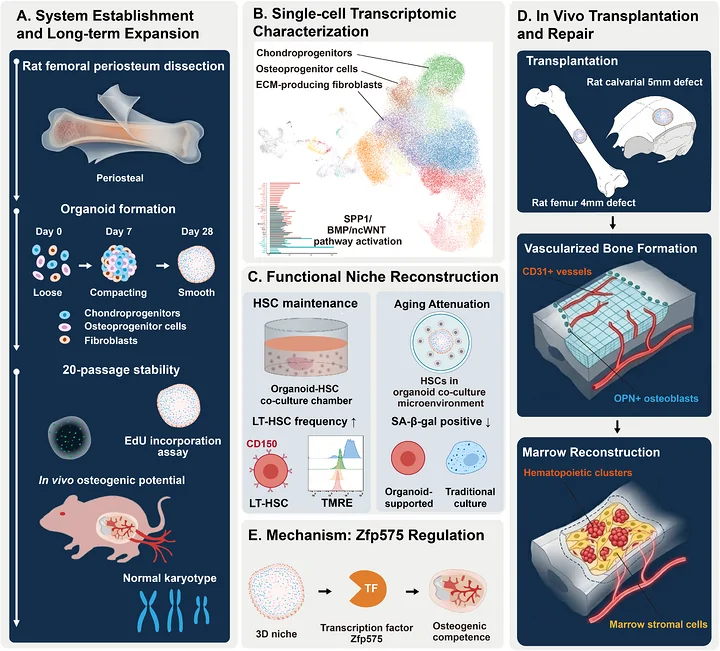

## 1 Introduction

The periosteum, a highly vascularized membrane enveloping most bone surfaces except at joints, plays a pivotal role in skeletal growth, repair, and regeneration due to its remarkable osteogenic potential [1, 2]. As early as 1867, Louis Xavier Ollier recognized the indispensable function of the periosteum in fracture healing, emphasizing that preserving its integrity was essential for successful bone repair [3]. Modern studies have reinforced this view, showing that periosteal removal severely compromises bone formation and neovascularization, highlighting its crucial role in skeletal regeneration [4]. Beyond serving as a structural envelope, the periosteum functions as a dynamic reservoir of skeletal stem cells (SSCs) with unique regenerative capacities [5, 6, 7, 8, 9, 10]. A landmark study by Debnath et al. highlighted the essential role of periosteal stem cells (PSCs) in intramembranous ossification, showing that disrupting their differentiation led to profound defects in bone healing [11]. Subsequent work identified a specialized subset of Mx1^+^αSMA^+^ periosteal SSCs exhibiting potent self‐renewal and colony‐forming capacities. These cells not only initiate osteogenesis upon injury but also support angiogenesis and regulate the local immune microenvironment [12]. Notably, unlike bone marrow‐derived SSCs, periosteal SSCs are rapidly activated in response to injury, emphasizing their frontline role in bone repair [13, 14]. While the inner cambium layer of the periosteum has traditionally been viewed as the major osteogenic compartment, recent evidence reveals that the outer fibrous layer also actively contributes to skeletal homeostasis and repair [15]. Beyond its role in repair, the periosteum may exert a protective function in pathological contexts‐for instance, periosteal stromal cells can fortify extracellular matrix barriers to restrict tumor invasion into adjacent bone tissue [16]. Collectively, these findings position the periosteum as a multifunctional organ‐an essential source of regenerative cells, a vascular and signaling hub, and a defensive interface critical for skeletal integrity.

Despite its central biological importance, faithfully reconstructing periosteal architecture and function in vitro remains a major challenge. Conventional two‐dimensional (2D) culture systems fail to preserve the native microenvironment and phenotypic diversity of periosteal cells. In contrast, organoid technologies have transformed tissue modeling by enabling three‐dimensional (3D) reconstruction of cellular organization and function [17, 18, 19, 20]. Recent efforts using periosteum‐derived progenitor cells have led to bone‐ and cartilage‐forming organoids that better mimic native tissue complexity. For instance, modular assembly of human periosteal‐derived cell (hPDC) micro‐organoids has enabled the generation of osteochondral constructs [21], while “callus organoids” recapitulate early endochondral ossification during fracture healing [22]. Bioengineering approaches combining melt electro‐written (MEW) polycaprolactone membranes with hPDC micro‐spheroids have yielded biohybrid sheets supporting chondrogenesis and ectopic bone formation [23]. Moreover, laser‐assisted bioprinting allows precise spatial organization of hPDC spheroids into multilayered cartilage‐like tissues with high cell viability [24], and biomimetic hydrogels integrating periosteal extracellular matrix promote robust osteogenesis in vivo [25]. These innovations highlight the regenerative potential of periosteal‐derived systems. However, current models are constrained by short‐term viability, limited expansion capacity, and incomplete recapitulation of native periosteal cell diversity and functionality.

Organoid systems across multiple tissues‐such as the intestine, brain, lung, and pancreas‐have set rigorous standards for in vitro modeling, emphasizing sustained self‐renewal, preservation of cellular heterogeneity, and long‐term physiological relevance [26, 27]. Guided by these principles, we developed a long‐term expandable periosteum organoid model that fulfills these criteria. Derived from primary periosteal cells, our organoids can be maintained for over 20 passages while preserving cellular heterogeneity and multilineage differentiation potential, as confirmed by single‐cell transcriptomic analysis and in vivo transplantation. Functionally, the organoid microenvironment supports HSC maintenance and mitigates aging‐associated decline, recapitulating niche‐like properties critical for skeletal health. Together, this work introduces a robust and physiologically relevant periosteum organoid system that combines long‐term expandability, cellular complexity, and functional competence, providing a versatile tool for modeling skeletal regeneration and tissue‐microenvironment interactions in vitro.

## 2 Results

### 2.1 Establishment of a Long‐Term Expandable Periosteal Organoid System With Preserved Growth Capacity and Genomic Stability

To establish stable periosteal organoids in vitro, we optimized existing protocols for organoid formation from adult stem cells by incorporating strategies from mesensphere culture systems [28, 29, 30]. Periosteal tissues were isolated from the femurs and tibias of 6‐week‐old Sprague‐Dawley (SD) rats using a periosteal elevator, a surgical instrument used to detach periosteum from the bone surface. Given the tight association between long bone periosteum and skeletal muscle, complete mechanical separation was challenging (Figure S1A), thus, enzymatic digestion was employed to generate a single‐cell suspension, followed by fluorescence‐activated cell sorting (FACS) to deplete Desmin^+^ muscle cells, thereby enriching for a highly viable, muscle‐depleted periosteal cell population (Figure S1B). These cells were then embedded in Matrigel for three‐dimensional (3D) culture (Figure 1A). An optimized mesenchymal expansion medium incorporating multiple growth and survival factors supported efficient organoid formation (Figure 1B, Video S1 and S2). Organoids derived from three independent batches exhibited consistent plating efficiency and day‐14 diameters, with minimal inter‐batch variation (Figure 1C,D). The average diameter decreased from ∼ 6000 µm at initiation to ∼ 3077 µm (range 2891–3290 µm) by day 14 (Figure S1C), and the diameter reduction ratio remained uniform across batches (Figure S1D). Unlike conventional spheroid organoids, periosteal organoids do not grow as compact cellular aggregates. Instead, cells progressively migrate toward the surface of the Matrigel dome and self‐organize into a thin periosteum‐like layer. During this process, cellular contraction and matrix remodeling lead to progressive compaction of the Matrigel dome, resulting in a reduction in dome diameter over time. To quantify organoid size, we defined the “dome size” as the diameter of each Matrigel droplet, with the diameter reduction ratio calculated as the diameter on a given day relative to its initial value at seeding. Live/dead staining followed by confocal Z‐stack imaging demonstrated that organoids maintained for up to 28 days showed high cell viability and preserved morphology, demonstrating long‐term structural integrity (Figure S1E,F).

**FIGURE 1:**
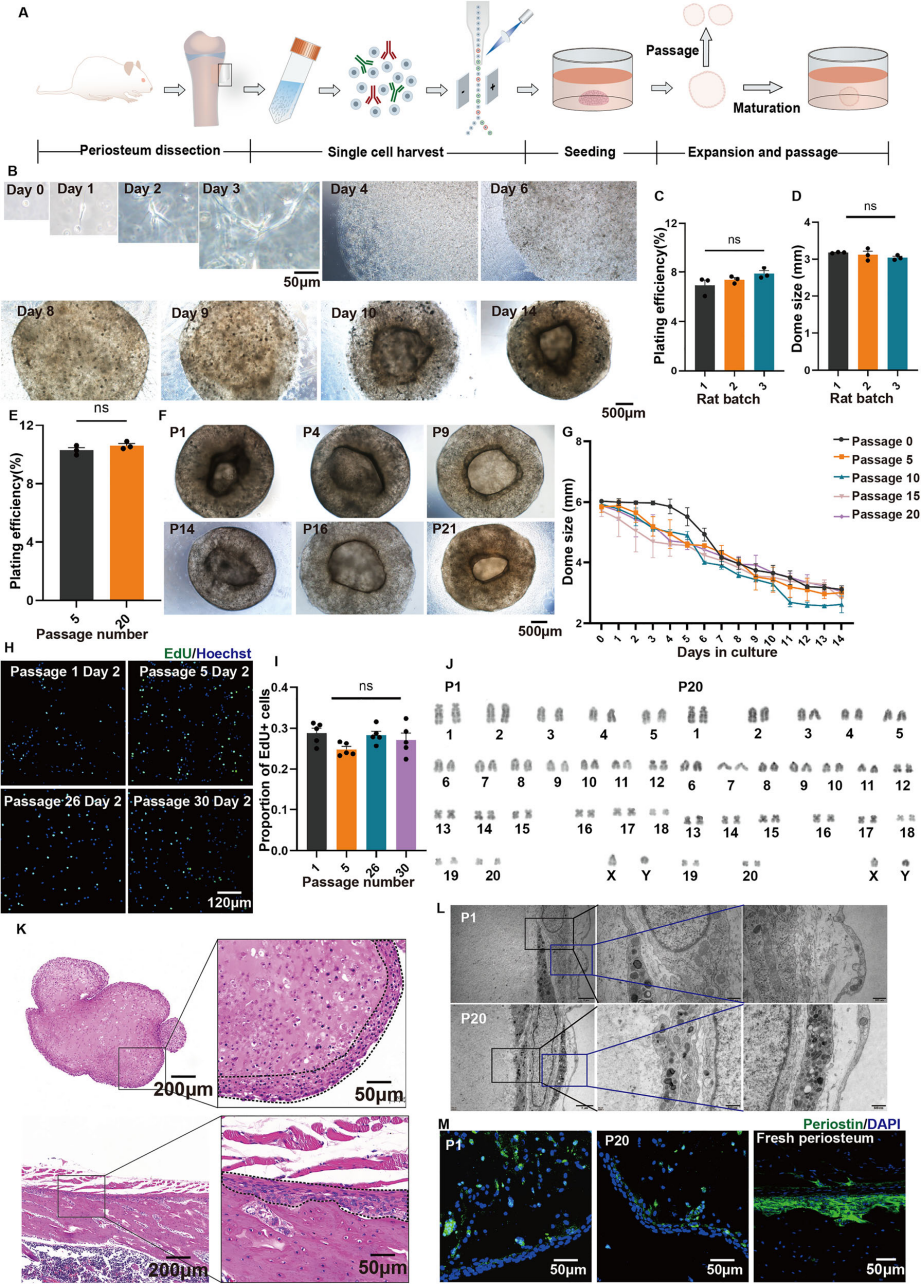
Establishment and characterization of long‐term expandable periosteal organoids. (A) Schematic depicting the protocol for generating periosteal organoids. (B) Representative phase‐contrast images of periosteal organoids from day 0 to day 14 (scale bars: 50 and 500 µm). (C) Plating efficiency of periosteal cells from three independent rat batches. Data are presented as mean ± SEM (*n* = 3). *P*‐values were calculated using one‐way ANOVA with Tukey's multiple comparisons test. ns, not significant. (D) Comparison of organoid diameters at day 14 from the three rat batches. Data are presented as mean ± SEM (*n* = 3). *P*‐values were calculated using one‐way ANOVA with Tukey's multiple comparisons test. ns, not significant. (E) Plating efficiency at passage 5 (P5) and passage 20 (P20). Data are presented as mean ± SEM, *n* = 3 per group. Statistical significance was assessed by two‐tailed unpaired Student's t‐test. ns, not significant. (F) Representative phase microscopy images of organoids during serial passaging, demonstrating stable morphology over long‐term culture (scale bar: 500 µm). (G) Growth curves (organoid diameter vs. culture time) for organoids at passages 0, 5, 10, 15, and 20. Data points represent mean ± SD from *n* ≥ 3 organoids per time point per passage. (H) Representative confocal images of organoids continuously passaged to P30 and pulse‐labeled with EdU for 16 h at day 2. EdU (green, laser 488), Hoechst (blue, laser 405) (Scale bar: 120 µm). (I) Quantification of EdU^+^ cell percentage across serial passages (P1‐P30). Data are presented as mean ± SEM (*n* = 5). *P*‐values were calculated using one‐way ANOVA with Tukey's multiple comparisons test. ns, not significant. (J) Representative karyotype images of P1 and P20 periosteal organoids, showing euploidy without structural aberrations. (K) Representative H&E staining images of a periosteal organoid and fresh periosteal tissue (scale bars: 50 and 200 µm). (L) Transmission electron microscopy (TEM) images of P1 and P20 organoids (scale bars: 2000 and 500 nm). (M) Immunofluorescence staining for periostin (green, laser 488) in P1 and P20 organoids; nuclei stained with DAPI (blue, laser 405). Scale bar: 50 µm.

Unlike epithelial organoids, which require 7–14 days to re‐plate [31, 32, 33], mesenchymal‐derived periosteal organoids can be re‐plated within 2–5 days and maintain undiminished growth for at least 20 passages, without observable declines in formation efficiency or day‐14 diameters, demonstrating robust long‐term expansion capacity (Figure 1E–G and Figure S1G). Real‐time imaging using the Incucyte system further showed that organoids from early (P5) and late (P20) passages displayed nearly identical growth kinetics (Figure S1H). Consistently, EdU incorporation assays revealed comparable proliferative activity between early and late passages (Figure 1H,I). Karyotype analysis revealed no detectable chromosomal abnormalities at either early (P1) or late (P20) passages, supporting the genetic stability of the periosteal organoids during long‐term expansion (Figure 1J).

To characterize organoid architecture and viability, hematoxylin and eosin (HE) staining showed that the organoids form a thin, multi‐layered cellular structure on the surface of the Matrigel dome, mimicking the native periosteum attached to bone matrix, with no necrotic core observed in the center (Figure 1K). Transmission electron microscopy revealed that both early‐ and late‐passage organoids maintained well‐preserved cellular ultrastructure, characterized by intact nuclear morphology, abundant intracellular organelles, and dense cytoplasmic organization (Figure 1L). Immunofluorescence staining showed comparable periostin expression in periosteal organoids at early (P1) and late (P20) passages, mirroring the native periosteal pattern: high expression in the inner cambium layer and low expression in the outer fibrous layer. This consistency with freshly isolated periosteal tissue supports preservation of periosteal identity during long‐term expansion (Figure 1M). Furthermore, immunofluorescence staining for extracellular matrix components revealed that the organoids secreted abundant fibronectin (FN1) and expressed connexin 43 (Cx43), indicating the formation of functional intercellular connections (Figure S1I,J). These findings support preservation of periosteal identity and matrix‐producing capacity during long‐term expansion. Collectively, these results demonstrate that periosteal organoid‐forming ability, proliferative capacity, structural integrity, and ECM composition are preserved across extended passaging. This scalability and genetic stability establish a reliable platform for mechanistic studies of periosteal biology.

### 2.2 Single‐Cell Analysis Reveals Long‐Term Transcriptional Stability of Periosteal Organoids

To systematically assess the stability of cellular states during long‐term in vitro expansion, we performed single‐cell RNA sequencing (scRNA‐seq) on freshly isolated periosteal cells and periosteal organoids collected from passage 0 (P0) through passage 20 (P20). After stringent quality control, we obtained a high‐quality dataset comprising 165 122 cells, enabling comprehensive longitudinal analysis of cellular composition and transcriptional dynamics.

Unsupervised clustering resolved eighteen transcriptionally distinct populations (Figure 2A). Based on established marker genes, these clusters were annotated as mesenchymal progenitor cells (Cluster 0; *Hmga1/2*, *Ccnd1*), chondroprogenitors (Cluster 1; *Col9a1/2/3*, *Col11a2*), G2/M‐phase cycling cells (Cluster 2; *Pttg1*, *Cdkn3*, *Ccnb2*), perivascular fibroblasts (Cluster 3; *Col4a1/2*, *Pdgfrb*, *Fap*) [34, 35], ECM‐producing fibroblasts (Cluster 4; *Sned1*, *Ptn*, *Chi3l1*) [36, 37, 38], osteoprogenitor cells (Cluster 5; *Runx2*, *Postn*) [6, 39], neuro‐like cells (Cluster 6; *Fat3*, *Rbfox1*, *Nrg1*), tendon‐like fibroblasts (Cluster 7; *Ccn2*, *Thbs2/4*), S‐ and M‐phase cycling cells (Clusters 8–9; *Top2a*, *Mki67*, *Cenpf*), endothelial cells (Cluster 10; *Cdh5*, *Pecam1*), mature ECM‐producing fibroblasts (Cluster 11; *Mfap5*, *Dpt*, *Fbn1*) [40, 41, 42, 43], macrophages (Cluster 12; *Csf1r*, *Aif1*), periosteal vascular smooth muscle cells (Cluster 13; *Acta2*, *Tagln*), immature B cells (Cluster 14; *Cd79a*, *Ighm*), activated fibroblasts (Cluster 15; *Atf3*, *Hmox1*, *Gadd45b*), proliferating chondrocytes (Cluster 16; *Col9a1/2/3*, *Mki67*), and neutrophils (Cluster 17; *S100a8/a9*, *Cxcr2*) (Figure 2A–C). Projection of fresh periosteal cells and periosteal organoids onto a shared UMAP space revealed extensive intermingling, indicating a high degree of global transcriptomic similarity (Figure 2B). When stratified by passage number, cells from P0 to P20 remained closely aligned with the fresh periosteal population, with only subtle passage‐associated gradients observed, suggesting minimal transcriptional drift during extended culture (Figure S2A). Analysis of cell‐type composition demonstrated that long‐term periosteal organoids robustly preserved the major mesenchymal compartments of native periosteum, which we define as the core mesenchymal lineages. These include mesenchymal progenitor cells, osteoprogenitors, chondroprogenitors, proliferating chondrocytes, ECM‐producing fibroblasts, mature ECM‐producing fibroblasts, and perivascular fibroblasts (Figure 2D). In contrast, non‐mesenchymal populations‐particularly immune and vascular lineages such as B cells, macrophages, and endothelial cells‐were progressively depleted during early passages, consistent with their dependence on niche‐specific in vivo cues not fully recapitulated in vitro. Notably, once established, the core periosteal mesenchymal compartments remained remarkably stable throughout long‐term expansion, as revealed by passage‐resolved longitudinal profiling (Figure 2E and Figure S2B). To validate the presence of immune lineages identified by scRNA‐seq at the protein level, we performed flow cytometric analysis of freshly isolated periosteal cells, passage 0 organoids, and passage 1 organoids. B cells (CD45^+^CD3^−^B220^+^) and macrophages (CD45^+^CD68^+^CD11b^+^) were readily detectable in freshly isolated periosteal cells, albeit at low frequencies (0.12% and 0.09%, respectively). Notably, both populations decreased substantially in passage 0 organoids (0.07% for B cells and 0.03% for macrophages) and became nearly undetectable by passage 1 (∼ 0.02% for both) (Figure S2C,D). These findings confirm the presence of these immune lineages at the protein level and demonstrate their rapid loss during early passaging, consistent with the scRNA‐seq data.

**FIGURE 2:**
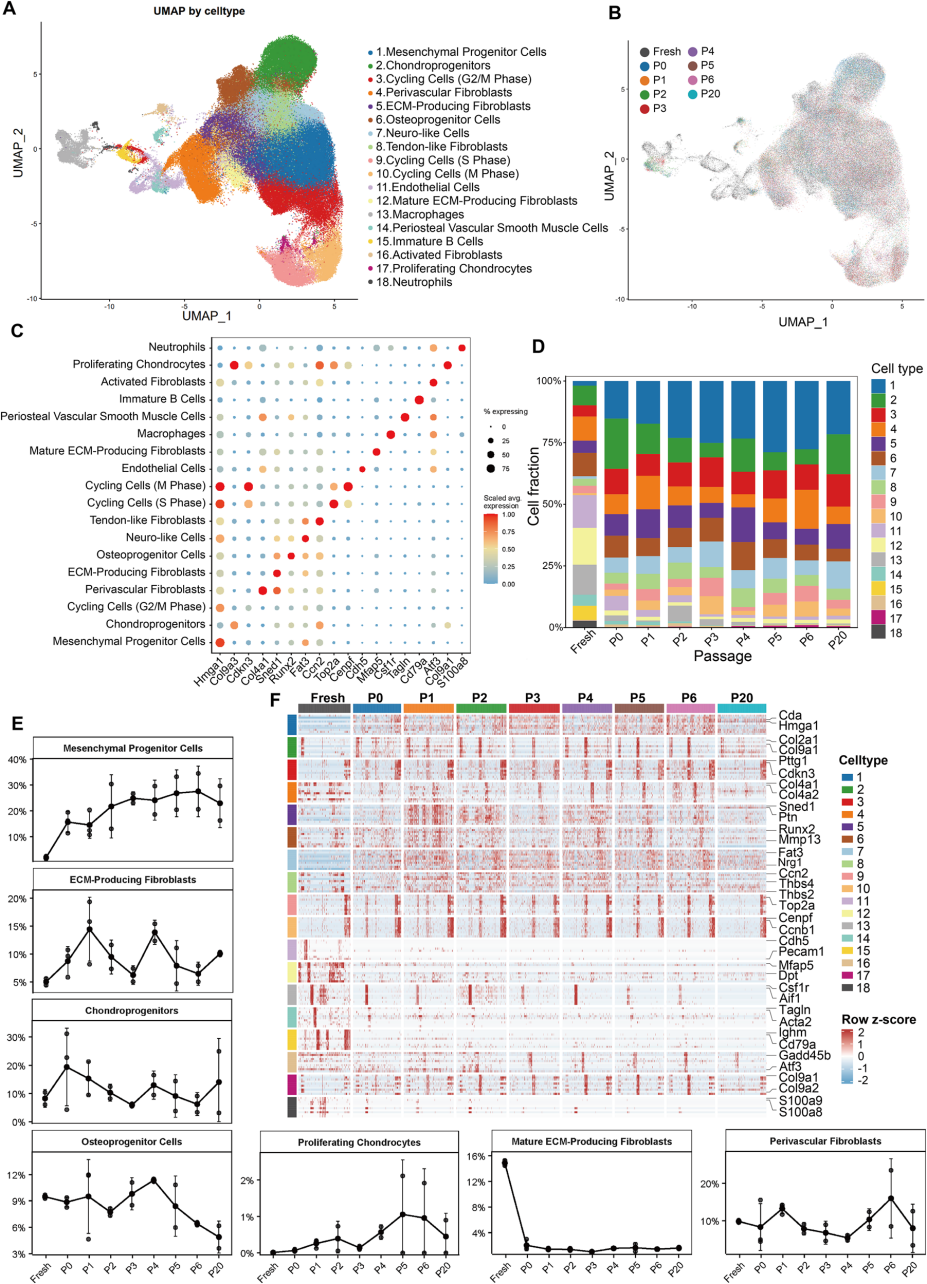
Single‐cell transcriptomic analysis reveals transcriptional and genomic stability of long‐term cultured periosteal organoids. (A) UMAP of the integrated single‐cell transcriptome showing 18 transcriptionally distinct cell clusters identified across fresh periosteum and organoid samples. (B) UMAP colored by sample origin (Fresh, P0‐P20), demonstrating global transcriptomic overlap between fresh tissue and organoids across passages. (C) Dot plot of canonical marker gene expression for each annotated cluster. Dot size represents the percentage of cells expressing the gene; color intensity indicates mean scaled expression level. (D) Stacked bar plot of cell‐type composition in fresh periosteum and organoids at each passage. (E) Passage‐resolved longitudinal profiles showing changes in cell‐type proportions across serial passaging (P0‐P20); data are presented as mean ± SD (*n* = 3). (F) Heatmap of normalized expression for cluster‐defining genes across samples (rows: genes; columns: samples/passage). Cluster color bar at right corresponds to cluster IDs in (A).

At the transcriptional level, heatmap analysis demonstrated a high concordance of cluster‐specific gene expression signatures between fresh periosteum and long‐term organoids across all major stromal lineages (Figure 2F). Consistently, cluster‐specific functional enrichment analysis revealed that each population retained lineage‐appropriate biological programs, including metabolic activity, biosynthetic processes, extracellular matrix organization, and tissue development pathways, further supporting the preservation of intrinsic cellular identities in vitro (Figure S3A). Intercellular communication networks were interrogated using Cell‐Chat analysis. Periosteal organoids retained a core signaling architecture characteristic of fresh periosteal tissue, while exhibiting a distinct shift in signaling emphasis. Pathways associated with periosteal regeneration and tissue patterning‐including SPP1, PTN, BMP, PDGF, FGF, and SLIT signaling‐were preserved or selectively enhanced in organoids. In contrast, inflammation‐ and immune‐associated pathways, such as TNF, CCL/CXCL, and complement signaling, were markedly attenuated compared with fresh periosteum (Figure S3B). To assess genomic stability during prolonged culture, we performed copy‐number variation (CNV) analysis across all passages. No detectable chromosomal abnormalities were observed in periosteal organoids (Figure S3C), in agreement with karyotype analysis (Figure 1J), confirming their genomic integrity during long‐term expansion. Collectively, these results establish periosteal organoids as a transcriptionally stable and genomically intact in vitro system that preserves core mesenchymal lineages and regenerative signaling networks of native periosteum during long‐term expansion.

### 2.3 Periosteal Organoids Engraft In Vivo and Generate Vascularized Bone and Marrow‐Like Microenvironments Across Extended Passaging

To evaluate the in vivo osteogenic and hematopoietic potential of periosteal organoids, freshly isolated periosteal cells (serving as a positive control) and organoids at different passages (P0, P5, P10, P15, P20) were subcutaneously transplanted into immunodeficient mice (Figure 3A). Micro‐CT analysis revealed that freshly isolated periosteal cells formed substantial mature mineralized bone tissue at 8 weeks post‐transplantation. Organoids at all passages showed comparable bone volume, bone mineral density, and BV/TV to freshly isolated periosteal cells, with no significant deterioration even at P20 (Figure 3B and Figure S4A). Histological examination further confirmed that freshly isolated periosteal cells formed mature bone matrix containing osteocyte lacunae and marrow‐like structures, which were indistinguishable from those formed by long‐term expanded organoids (Figure 3C and Figure S4B,C). It is noteworthy that P26 organoids, after cryopreservation and subsequent recovery six months later, retained their osteogenic capacity in vivo and are capable of reconstructing marrow‐like structures in grafts (Figure S4D).

**FIGURE 3:**
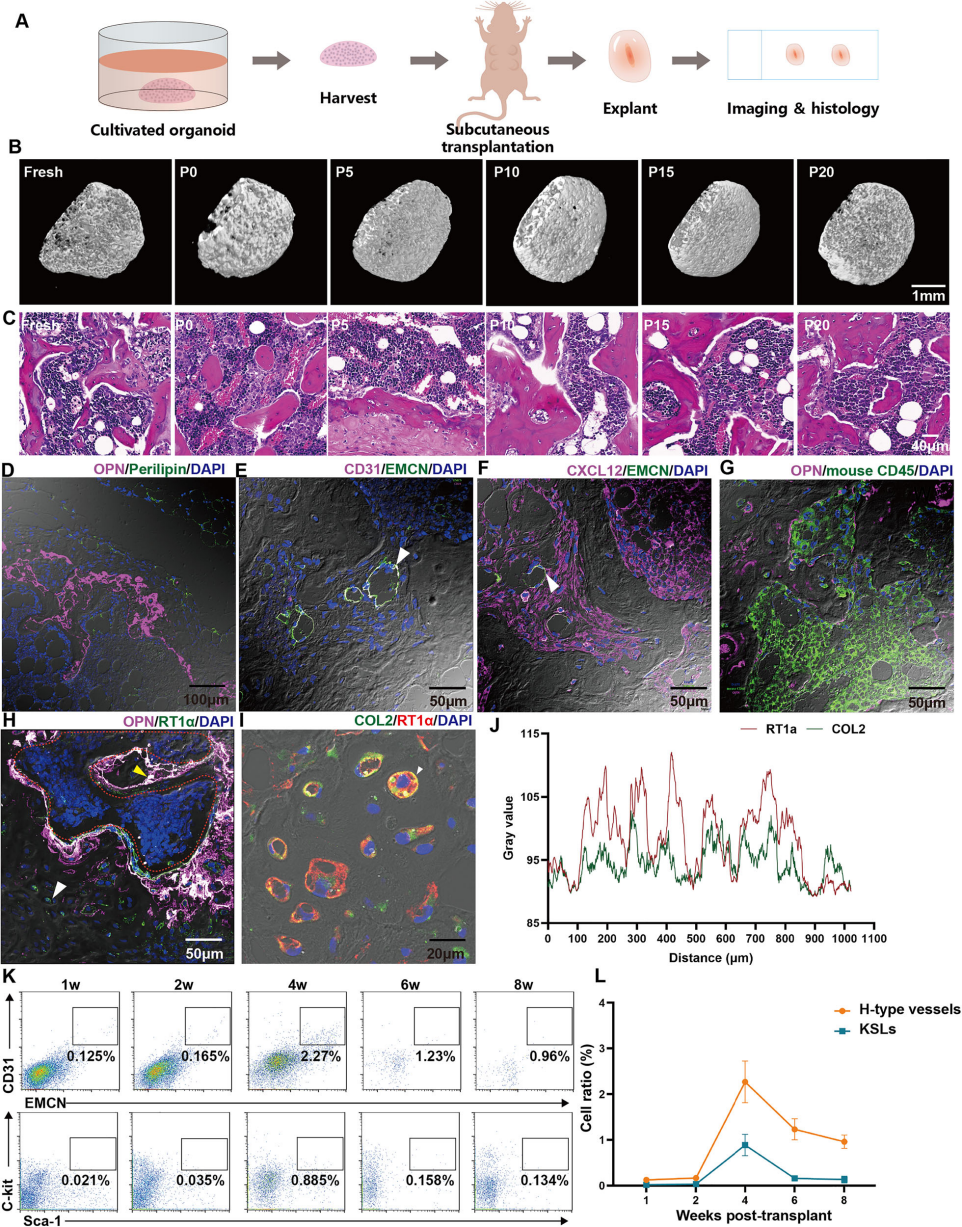
In vivo engraftment and functional maturation of periosteal organoids. (A) Schematic of the subcutaneous organoid transplantation assay in immunodeficient mice. (B) Representative 3D micro‐CT reconstruction images of 8‐week grafts derived from fresh periosteal cells or organoids at different passages. (C) H&E staining of 8‐week grafts from fresh periosteum and organoids at different passages. Scale bar: 40 µm. (D) Immunofluorescence for Perilipin (green, laser 488) and OPN (purple, laser 555) in 8‐week grafts. Scale bar: 100 µm. (E) Immunofluorescence for EMCN (green, laser 488) and CD31 (purple, laser 555) in 8‐week grafts. White arrow indicates CD31^+^EMCN^+^ endothelial structures forming luminal‐like arrangements. Scale bar: 50 µm. (F) Immunofluorescence staining for EMCN (green, laser 488) and CXCL12 (purple, laser 555) in 8‐week grafts. White arrow indicates EMCN^+^ cells forming luminal‐like arrangements. Scale bar: 50 µm. (G) Immunofluorescence for host‐derived mouse CD45 (green, laser 488) and OPN (purple, laser 555) in 8‐week grafts. Scale bar: 50 µm. (H) Immunofluorescence staining for donor‐specific RT1α (red, 647 nm) and OPN (purple, 555 nm) in 8‐week grafts. Newly formed bone (OPN^+^) is indicated by yellow arrows, cells with cartilage morphology by white arrows, and the marrow cavity is outlined by a red dashed line. Scale bar: 50 µm. (I) Immunofluorescence staining for donor‐specific RT1α (red, 647 nm) and COL2 (green, 488 nm) in 2‐week grafts. Newly formed cartilage (COL2^+^) is indicated by white arrows. Scale bar: 50 µm. (J) Representative plot profile analysis of RT1a and COL2 fluorescence intensities. (K) Representative flow cytometric gating plots showing CD31^+^EMCN^+^ H‐type vessels (top) and LSK cells (bottom) in grafts at indicated time points post‐transplantation. (L) Quantification by flow cytometry of CD31^+^EMCN^+^ H‐type vessels and c‐Kit^+^Sca‐1^+^Lin^−^ (LSK) cells in grafts at indicated time points post‐transplantation. Data are presented as mean ± SD (*n* = 3).

Perilipin immunostaining and Oil Red O staining confirmed that vacuolar structures within the grafts were not adipocytes but likely residual Matrigel (Figure 3D and Figure S4E). Mature grafts contained abundant marrow‐like cavities populated by hematopoietic cells, accompanied by EMCN^+^ endothelial structures forming luminal‐like arrangements (Figure 3E). CXCL12‐expressing stromal cells were readily detected within these regions, indicating the establishment of a bone marrow‐like microenvironment associated with hematopoietic cell support (Figure 3F). Mouse‐specific CD45 staining revealed that the majority of cells within the marrow cavity of the ectopic bone originated from the host (Figure 3G). In contrast, RT1a staining revealed that the newly formed bone (OPN^+^, indicated by yellow arrow) and cells with cartilage morphology (indicated by white arrow) were derived from the transplanted organoids, confirming their donor origin (Figure 3H). To further verify the cartilage origin, co‐immunofluorescence staining for COL2 and RT1a was performed on 2‐week grafts, which confirmed that the majority of COL2^+^ cartilage was indeed donor‐derived (Figure 3I). Plot profile analysis revealed that the fluorescence intensities of RT1a and COL2 exhibited parallel fluctuation patterns along the measured distance, further supporting their spatial colocalization (Figure 3J). Colocalization analysis between RT1a (graft marker) and COL2 (cartilage marker) was performed on three random fields using Fiji Coloc 2 with Costes thresholding. Manders’ tM1 (COL2^+^ area overlapping with RT1a^+^) ranged from 0.61 to 0.78 (mean ± SD: 0.67 ± 0.10, *n* = 3), with all values exceeding the 0.5 significance threshold. Pearson's R (above‐threshold) ranged from 0.36 to 0.47 (mean ± SD: 0.41 ± 0.06, *n* = 3), indicating a moderate positive correlation. These data confirm that the majority of newly formed COL2^+^ cartilage was derived from RT1a^+^ graft cells, with modest regional heterogeneity consistent with ongoing chondrogenic maturation.

Temporal analysis of grafts at 1, 2, 4, and 8 weeks revealed dynamic tissue maturation (Figure S5A). At 1 week, grafts showed abundant COL2^+^COL10^+^ cells indicative of prehypertrophic chondrocytes (Figure S5B–F). At 2 weeks, the grafts contained large numbers of cartilage cells with high COL10 expression, and COL2 expression was markedly decreased relative to 1‐week grafts (Figure S5G–K). By 4 weeks, Safranin O/Fast Green staining demonstrated newly formed bone matrix (blue‐green) and collagen or unmineralized matrix (red), while H&E showed numerous osteoblasts and embedded osteocytes, consistent with active bone formation (Figure S5L–P). At 8 weeks, dense mineralized bone was evident (Figure S5Q–T).

To assess temporal dynamics of marrow‐like structures, we analyzed c‐Kit^+^Sca‐1^+^Lin^−^ (LSK) hematopoietic stem/progenitor cells and CD31^+^EMCN^+^ endothelial cells at multiple time points from 1 to 8 weeks. Both populations peaked at 4 weeks (LSK: 1.97%; CD31^+^EMCN^+^: 6.79%) and then gradually declined thereafter (Figure 3K,L). Flow cytometry further demonstrated the presence of hematopoietic progenitors within grafts at 8 weeks post‐transplantation. Using CD34 and CD16/32, GMP, CMP, and MEP populations were all detectable, indicating maintenance of diverse hematopoietic progenitor subsets (Figure S5U).

To further distinguish whether the marrow‐like structures within the grafts represented inflammatory infiltration or genuine bone marrow, we analyzed the temporal changes of host immune cells and compared the morphology of 8‐week grafts with normal rat bone marrow. Flow cytometry at 2 days, 1 week, 2 weeks, 4 weeks, 6 weeks, and 8 weeks revealed that neutrophils infiltrated prominently at 2 days and macrophages at 1 week, but both declined to very low levels by 4 weeks (< 0.03% of total cells), indicating a transient and resolving host immune response (Figure S5V,W). In contrast, KSL cells were maintained at approximately 0.1% at 6 weeks and afterward, and MEP, GMP, and CMP remained detectable at 8 weeks (Figure 3K and Figure S5U), supporting the establishment of a stable hematopoietic microenvironment. Morphological comparison of HE‐stained 8‐week graft sections with normal rat bone marrow confirmed that the graft‐derived marrow cavities contained typical bone marrow constituents, including multilineage hematopoietic cells (erythroid, granulocytic, lymphoid precursors), megakaryocytes, osteoblasts lining the bone surface, as well as sinusoids containing mature erythrocytes (Figure S5X). Based on these histological and cytometric features, we define “marrow‐like structures” in this study as cavity‐like areas within the graft that contain multilineage hematopoietic cells (erythroid, granulocytic, lymphoid precursors), megakaryocytes, sinusoids containing mature erythrocytes, together with detectable GMP/CMP/MEP progenitors by flow cytometry. This combination of features recapitulates key aspects of the native bone marrow microenvironment. These observations collectively indicate that periosteal organoids can engraft, differentiate, and partially reconstitute a vascularized bone and marrow microenvironment in vivo.

### 2.4 Periosteal Organoids Provide a Niche‐Like Microenvironment That Supports HSC Survival and Preserves a Slow‐Cycling State Ex Vivo

Building on our in vivo transplantation studies demonstrating that periosteal organoids can generate marrow‐like cavities, we next asked whether these organoids also provide a functional niche capable of supporting HSCs during extended ex vivo culture. To address this question, we established an experimental workflow in which FACS‐purified primary mouse HSCs (Figure S6A) were introduced into periosteal organoid cultures and subsequently re‐isolated for longitudinal assessment of viability, phenotype, and molecular status over 7, 14, and 28 days (Figure 4A). Parallel cultures were performed using co‐culture medium or standard HSC medium as controls. Using a trans‐well assay, we first evaluated the chemotactic and supportive effects of periosteal organoids on HSCs. Organoids attracted the highest number of viable HSCs, whereas co‐culture medium provided only modest improvement over baseline culture conditions, indicating that intact organoids offer superior niche support (Figure 4B). Consistently, single‐cell RNA sequencing revealed a niche signaling program that is maintained over time within periosteal organoids, with coordinated expression of canonical HSC‐maintenance factors preserved from freshly isolated samples through successive passages (Figure 4C and Figure S6B).

**FIGURE 4:**
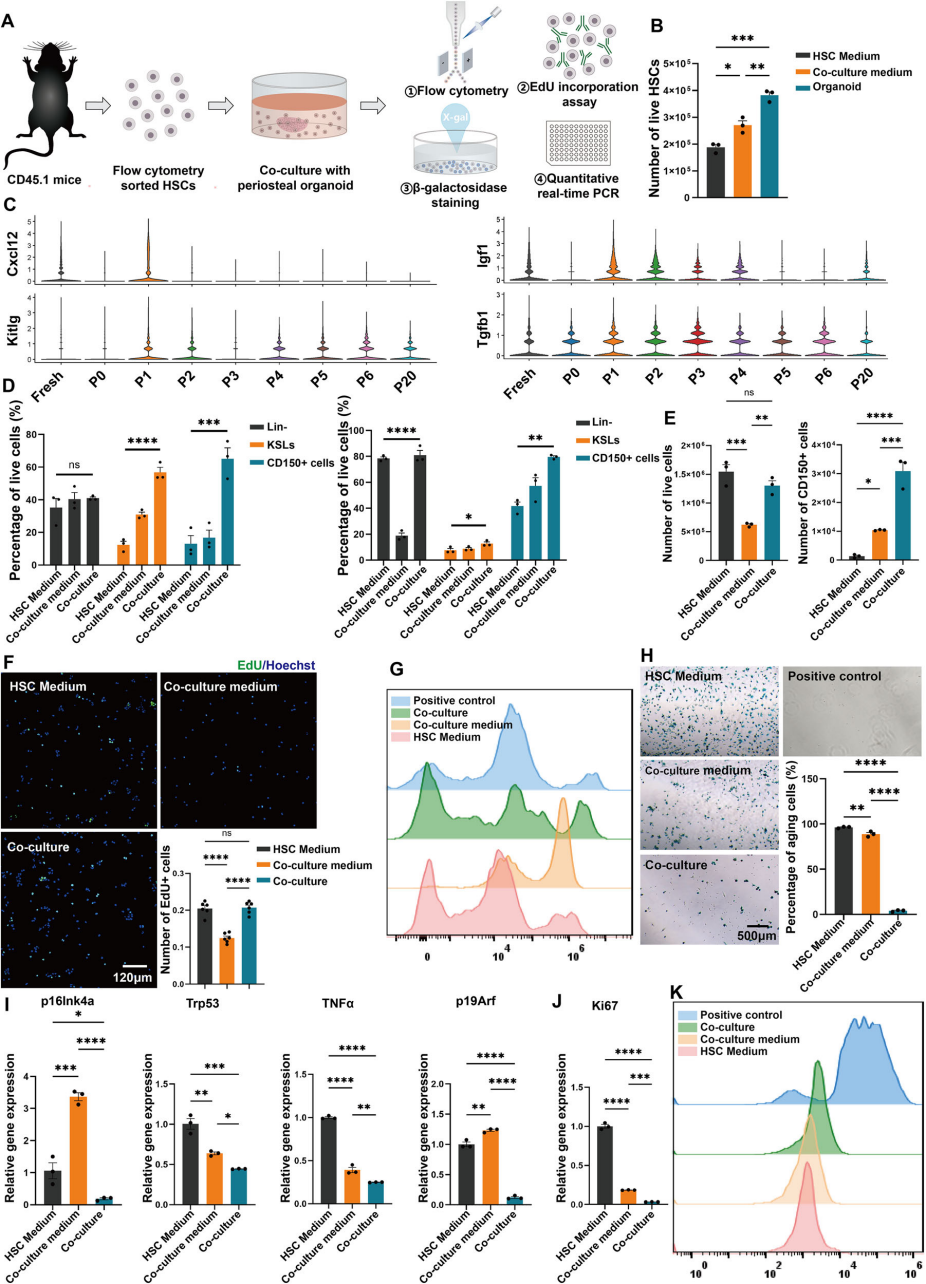
Periosteal organoids provide a functional ex vivo niche supporting HSC maintenance and delaying functional aging. (A) Schematic of the HSC co‐culture experimental workflow. (B) Trans‐well assay quantifying HSC migration toward periosteal organoids or co‐culture medium. Analysis was restricted to hematopoietic cells; organoid cells were excluded. Cell counts are normalized to the standard HSC medium control. Data are presented as mean ± SEM (*n* = 3). *P*‐values were calculated using one‐way ANOVA with Tukey's multiple comparisons test. ^*^ *P* < 0.05, ^**^ *P* < 0.01, ^***^ *P* < 0.001. (C) Violin plots of key HSC‐supportive factor expression in fresh periosteum and organoids at indicated passages from scRNA‐seq data. (D) Flow cytometry analysis of phenotypic HSC maintenance (LSK and CD150^+^ subsets) after 7 (left) and 14 days (right) of culture under indicated conditions. Data are presented as mean ± SEM (*n* = 3). *P*‐values were calculated using one‐way ANOVA with Tukey's multiple comparisons test. ns, not significant, ^*^ *P* < 0.05, ^**^ *P* < 0.01, ^***^ *P* < 0.001, ^****^ *P* < 0.0001. (E) Total cell output and number of CD150^+^ cells generated from 35 input HSCs after 14 days of culture. Data are presented as mean ± SEM (*n* = 3). *P*‐values were calculated using one‐way ANOVA with Tukey's multiple comparisons test. ns, not significant, ^*^ *P* < 0.05, ^**^ *P* < 0.01, ^***^ *P* < 0.001, ^****^ *P* < 0.0001. (F) Representative confocal images and quantification of EdU pulse‐labeled HSCs after 14 days of culture (EdU, green, laser 488; Hoechst, blue, laser 405). Scale bar: 120 µm. Data are presented as mean ± SEM (*n* = 6). *P*‐values were calculated using one‐way ANOVA with Tukey's multiple comparisons test. ns, not significant, ^****^ *P* < 0.0001. (G) Flow cytometry analysis of intracellular reactive oxygen species (ROS) levels in HSCs after 14 days under different culture conditions. (H) Senescence‐associated β‐galactosidase (SA‐β‐gal) staining of HSCs after 28 days of culture. Representative images and quantification of SA‐β‐gal ^+^ cells. Data are presented as mean ± SEM (*n* = 3). *P*‐values were calculated using one‐way ANOVA with Tukey's multiple comparisons test. ^**^ *P* < 0.01, ^****^ *P* < 0.0001. Scale bar: 500 µm. (I) Quantitative RT‐PCR analysis of senescence‐related gene expression (*p19Arf*, *p16Ink4a*, *Tnf*, *Trp53*) in cells derived from HSCs after 28 days. Data are normalized to *Gapdh* and presented as mean ± SEM (*n* = 3). *P*‐values were calculated using one‐way ANOVA with Tukey's multiple comparisons test. ^*^ *P* < 0.05, ^**^ *P* < 0.01, ^***^ *P* < 0.001, ^****^ *P* < 0.0001. (J) Quantitative RT‐PCR analysis of *Mki67* expression in cells derived from HSCs after 28 days. Data are normalized to *Gapdh* and presented as mean ± SEM (*n* = 3). *P*‐values were calculated using one‐way ANOVA with Tukey's multiple comparisons test. ^***^ *P* < 0.001, ^****^ *P* < 0.0001. (K) Flow cytometry analysis of mitochondrial membrane potential (TMRE) in HSCs after 28 days of culture (representative histograms shown).

We next examined HSC survival and phenotypic preservation during extended culture. At day 7, organoid co‐culture preserved both LSK and CD150^+^ subsets more effectively than either co‐culture medium or standard medium. Although LSK viability decreased across all groups by day 14, the organoid‐supported niche consistently preserved the largest fraction of viable cells, with CD150^+^ HSCs showing similar trends (Figure 4D). Assessment of total viable single cells revealed that co‐culture maintained overall cell numbers comparable to standard medium, whereas co‐culture medium led to a pronounced reduction. Analysis of the primitive CD150^+^ population confirmed that organoid co‐culture uniquely preserved these cells over time (Figure 4E). To assess functional maintenance, we evaluated proliferation, oxidative stress, and aging phenotypes. EdU incorporation at day 14 indicated that organoid co‐cultured HSCs retained proliferation rates comparable to standard medium and substantially higher than those exposed to co‐culture medium (Figure 4F). Intracellular ROS measurements revealed elevated oxidative stress in co‐culture medium‐cultured HSCs, whereas organoid co‐culture shifted ROS distributions toward a slow‐cycling profile resembling that observed in standard medium (Figure 4G). After 28 days, senescence‐associated β‐galactosidase staining revealed markedly reduced HSC senescence exclusively in the organoid co‐culture (Figure 4H), accompanied by significantly decreased expression of p16, p19, p53, and the SASP cytokine TNFα (Figure 4I). Although Ki67 expression was also reduced (Figure 4J), this coordinated downregulation is indicative of a slow‐cycling state rather than proliferative exhaustion, consistent with the preservation of a primitive HSC phenotype. Flow cytometric analysis of Ki‐67 and DAPI co‐staining further demonstrated that HSCs co‐cultured with periosteal organoids exhibited a significantly higher proportion of cells in G0 phase (approximately 20.1%) compared to those cultured in standard medium (approximately 6.12%) or co‐culture medium (approximately 8.59%) (Figure S6C). We next assessed mitochondrial membrane potential using TMRE staining. Freshly isolated HSCs exhibited a characteristic TMRE distribution, whereas HSCs cultured in co‐culture medium showed a pronounced leftward shift, indicative of reduced mitochondrial activity. In contrast, HSCs directly co‐cultured with periosteal organoids displayed higher TMRE signals with a notably narrower distribution, suggesting a more homogeneous and regulated metabolic state (Figure 4K).

Despite these beneficial effects on HSC phenotype and stress markers ex vivo, we next asked whether this translated into long‐term functional reconstitution in vivo. We performed competitive transplantation assays by injecting 150 HSCs (CD45.1) that had been co‐cultured with periosteal organoids for 14 days together with 2 × 10^5^ competitor whole bone marrow cells (CD45.1^+^CD45.2^+^) into lethally irradiated CD45.2 recipients. Peripheral blood chimerism examined at 4 weeks post‐transplantation was below 5% under all conditions tested (*n* = 5), indicating no detectable multilineage reconstitution (Figure S6D). Therefore, while periosteal organoids delay HSC functional aging and maintain a primitive phenotype ex vivo, they do not preserve long‐term repopulating capacity under the current culture conditions. Together, these results indicate that periosteal organoids establish a durable ex vivo niche that supports HSC survival, preserves a slow‐cycling state, and delays functional aging, as evidenced by preserved phenotype, lower oxidative stress, and reduced senescence. However, competitive transplantation revealed that this maintenance does not extend to long‐term multilineage reconstitution in vivo.

### 2.5 ZFP575 is Selectively Maintained in 3D Organoids and is Required for Preserving In Vivo Osteogenic Competence

Periosteal tissues rapidly lose their in vivo differentiation potential under conventional 2D culture conditions (Figure 5A). To identify molecular factors that sustain functional integrity in 3D periosteal organoids, we performed bulk RNA‐seq comparing 2D‐cultured periosteal cells to 3D periosteal organoids. Differential expression analysis revealed a set of transcription factors selectively preserved in 3D organoids but markedly downregulated in 2D culture (Figure 5B), suggesting their potential roles in maintaining periosteal identity under three‐dimensional conditions. Among these candidates, *Zfp575* showed a consistent and pronounced loss of expression during 2D culture, while remaining stably expressed in 3D organoids, as confirmed by qRT‐PCR (Figure 5C and Figure S7A). Cell‐type‐resolved expression analysis further revealed that *Zfp575* was uniquely enriched in osteo‐progenitor populations, which are directly responsible for lineage commitment and bone formation in vivo (Figure S7B). We therefore prioritized *Zfp575* for functional interrogation. PCR and western blotting confirmed efficient knockdown of *Zfp575* in periosteal organoids (Figure S7C–E). *Zfp575* knockdown did not impair in vitro organoid formation or growth kinetics through day 15 (no significant difference in dome diameter; Figure 5D,E and Figure S7F–H). However, following transplantation, *Zfp575*‐deficient organoids exhibited a marked loss of in vivo osteogenic potential: micro‐CT and histology showed significantly reduced bone volume (BV), bone volume fraction (BV/TV), and bone mineral density (BMD) compared with controls (Figure 5F–I), with corresponding decreases in newly formed bone and mineralized matrix on histology (Figure 5J and Figure S7I,J). Together, these data indicate 3D culture preserves lineage‐specific transcriptional programs and identify ZFP575 as a non‐redundant regulator required to maintain periosteal progenitor competence for in vivo osteogenesis.

**FIGURE 5:**
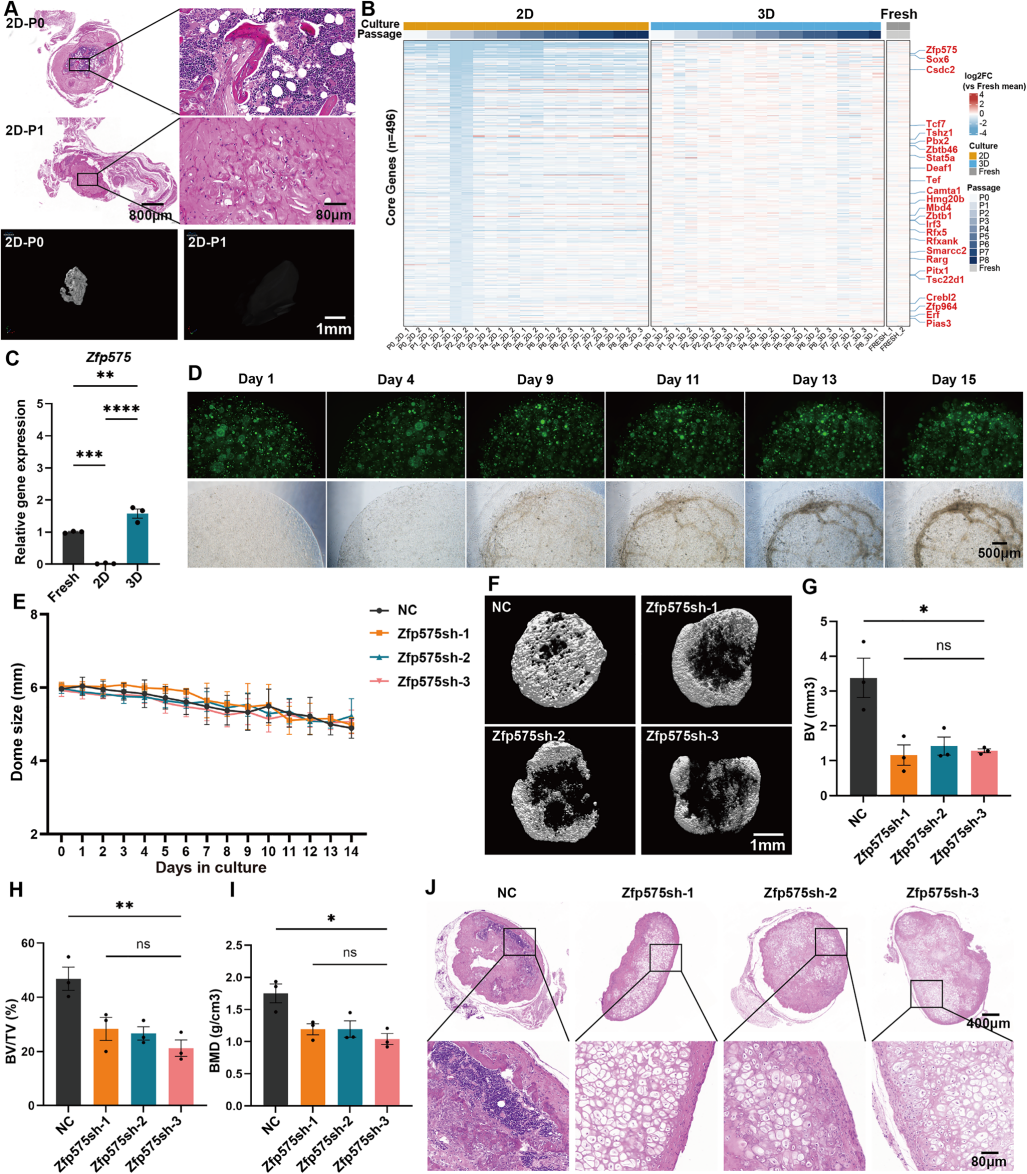
ZFP575 is selectively maintained in 3D periosteal organoids and is required for their in vivo osteogenic competence. (A) Representative H&E staining and micro‐CT reconstruction of grafts 8 weeks after subcutaneous transplantation of 2D‐cultured periosteal cells (P0, P1) into nude mice. Scale bars: 800 µm (H&E, left), 80 µm (H&E, right), 1 mm (micro‐CT). (B) Heatmap comparing gene expression profiles of fresh periosteum, 2D‐cultured cells, and 3D organoids. (C) Quantitative RT‐PCR analysis of *Zfp575* expression in fresh periosteum, 2D‐cultured cells, and organoids. Data are presented as mean ± SEM (*n* = 3). *P*‐values were calculated using one‐way ANOVA with Tukey's multiple comparisons test. ^**^ *P* < 0.01, ^***^ *P* < 0.001, ^****^ *P* < 0.0001. (D) Figure of GFP‐positive cells in *Zfp575*sh‐1 organoids during 15 days of puromycin selection. (E) Growth curves of organoid diameters over 14 days (*n* ≥ 3 organoids per time point; mean ± SD). (F) Micro‐CT reconstruction of grafts 8 weeks after transplantation of control (NC) or *Zfp575*‐knockdown organoids. Scale bar: 1 mm. (G–I) Quantitative micro‐CT analysis of bone mineral density (BMD) (G), bone volume (BV) (H), and bone volume fraction (BV/TV) (I) in 8‐week grafts. Data are presented as mean ± SEM (*n* = 3). *P*‐values were calculated using one‐way ANOVA with Tukey's multiple comparisons test. ns, not significant, ^*^ *P* < 0.05, ^**^ *P* < 0.01. (J) H&E staining of 8‐week grafts from NC and *Zfp575*‐knockdown organoids. Scale bars: 400 µm (up), 80 µm (bottom).

### 2.6 Engineered Periosteal Organoids Promote Bone Regeneration and Structural Repair Across Distinct Skeletal Defect Models

To determine whether engineered periosteal organoids retain regenerative function in vivo after long‐term expansion, we evaluated their therapeutic efficacy in both long‐bone and calvarial defect models. Organoids derived from long‐bone periosteum were implanted at early (P5) and late (P25) passages, with one organoid placed per defect, and 40 µL of blank Matrigel served as a control. At 8 weeks, micro‐CT analyses revealed increased mineralized tissue formation in organoid‐treated long‐bone defects compared with controls (Figure 6A). Quantitative analysis showed increased BMD and BV in organoid‐treated groups relative to controls (Figure 6B,C). Regenerative outcomes were comparable between P5 and P25 organoids, suggesting preserved in vivo functionality after extended expansion. Histological evaluation revealed that the 4 mm diameter femoral defects in the organoid‐transplanted group were almost completely bridged by newly formed bone, in contrast to the control group, which exhibited limited union. H&E, Masson's trichrome staining, and Safranin O/Fast Green staining of the grafted area demonstrated abundant newly formed trabecular bone, with visible osteocytes embedded in the lacunae. The graft‐host interface showed seamless integration with no fibrous encapsulation, indicating excellent biocompatibility and osseointegration of the organoid‐derived constructs (Figure 6D and Figure S8A,B). Immunofluorescence staining for OPN further indicated the presence of functionally active osteoblasts and confirmed the ongoing matrix mineralization at the protein level (Figure 6E). To assess vascularization, we performed CD31 immunofluorescence staining on femoral defect sections. The organoid‐treated group showed CD31^+^ endothelial structures within the newly formed bone, providing direct evidence of vascularized bone regeneration (Figure 6F).

**FIGURE 6:**
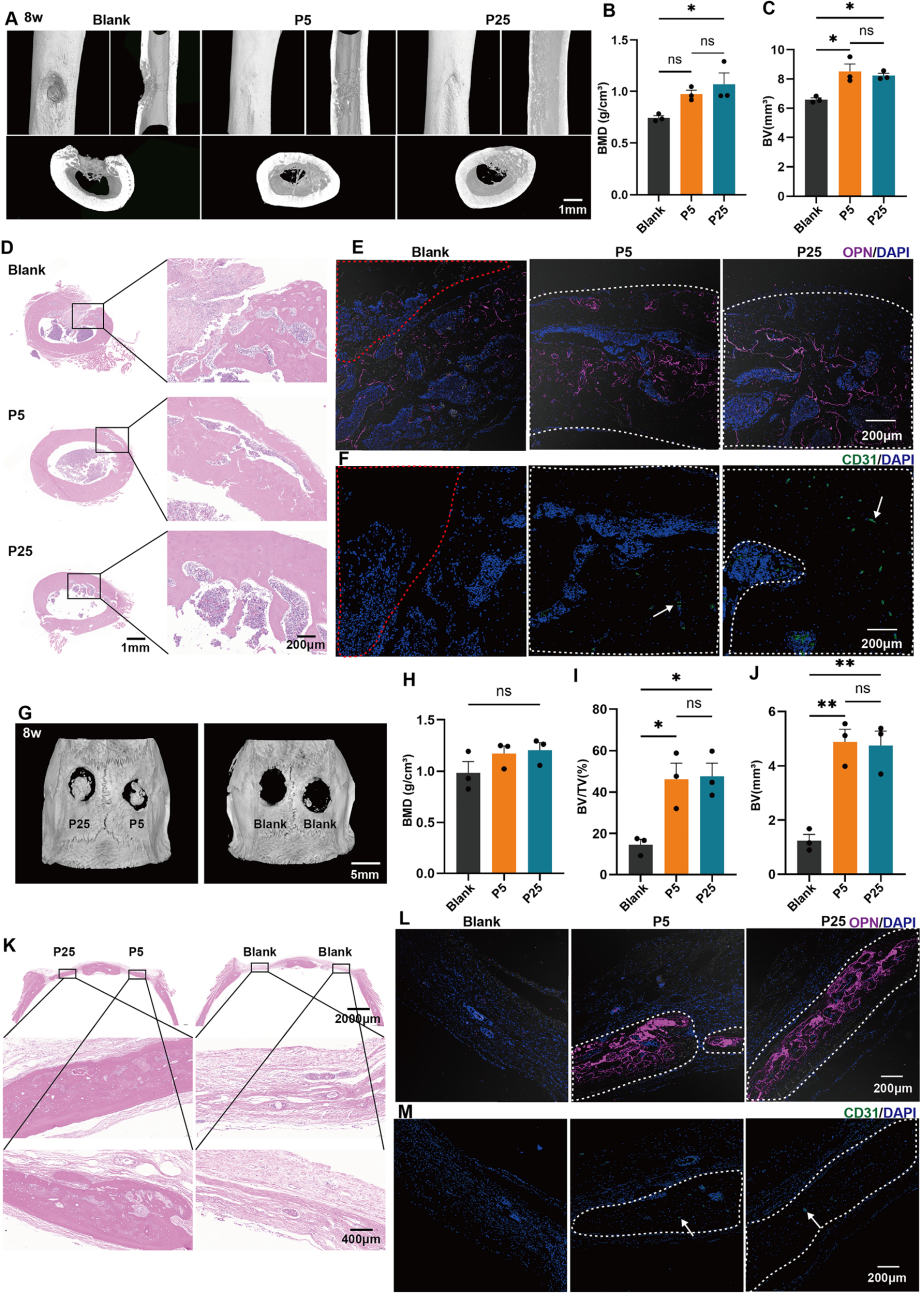
Engineered periosteal organoids promote functional bone regeneration in critical‐sized defect models. (A) Representative micro‐CT reconstruction of rat femoral defects 8 weeks after transplantation with Matrigel, P5 organoids, or P25 organoids. Scale bar: 1 mm. (B, C) Quantitative analysis of BMD (B) and BV (C) in femoral defects at 8 weeks. Data are presented as mean ± SEM (*n* = 3 biological replicates). *P*‐values were calculated using one‐way ANOVA with Tukey's multiple comparisons test. ns, not significant, ^*^ *P* < 0.05. (D) H&E staining of femoral defect areas at 8 weeks. Scale bars: 1 mm (left), 200 µm (right). (E) Immunofluorescence staining for OPN (purple, laser 555) of femoral defect areas at 8 weeks. Red dashed lines outline the unrepaired defect area, and white dashed lines indicate the newly formed bone region. Scale bar: 200 µm. (F) Immunofluorescence staining for CD31 (green, 488 nm) in femoral defect areas at 8 weeks. Red dashed lines outline the unrepaired defect area, and white dashed lines indicate the newly formed bone region. Scale bar: 200 µm. (G)Micro‐CT reconstruction of rat calvarial defects 8 weeks after transplantation. Scale bar: 5 mm. (H–J) Quantitative analysis of BMD (H), BV/TV (I), and BV (J) in calvarial defects at 8 weeks. Data are presented as mean ± SEM (*n* = 3 biological replicates). *P*‐values were calculated using one‐way ANOVA with Tukey's multiple comparisons test. ns, not significant, ^*^ *P* < 0.05, ^**^ *P* < 0.001. (K) H&E staining of calvarial defect areas at 8 weeks. Scale bars: 2000 µm (top), 400 µm (bottom). (L) Immunofluorescence staining for OPN (purple, laser 555) of calvarial defect areas at 8 weeks. White dashed lines indicate the newly formed bone region. Scale bar: 200 µm. (M) Immunofluorescence staining for CD31 (green, 488 nm) in calvarial defect areas at 8 weeks. White dashed lines indicate the newly formed bone region, and white arrows indicate CD31^+^ endothelial structures. Scale bar: 200 µm.

To assess whether regenerative activity extends across skeletal contexts, we next tested long‐bone‐derived organoids in a calvarial defect model. Micro‐CT reconstruction showed new bone formation in organoid‐treated groups, whereas blank Matrigel‐treated defects exhibited limited spontaneous repair (Figure 6G and Figure S8C). Quantitative measurements demonstrated significant increases in BV/TV and BV in both P5 and P25 groups relative to controls, with no significant difference between passages (Figure 6H–J and Figure S8D–F). These results indicate that long‐term expanded organoids retain regenerative competence across anatomically distinct bone environments. Histological evaluation confirmed enhanced regeneration following organoid implantation. H&E staining revealed extensive new bone formation in treated defects, whereas controls were primarily occupied by fibrous tissue (Figure 6K and Figure S8G). Newly formed tissue displayed organized bone‐like architecture containing marrow‐like spaces, consistent with progressive tissue maturation. Masson's trichrome and Safranin O/Fast Green staining supported active extracellular matrix remodeling during repair (Figure S8H,I). Immunofluorescence staining for OPN further validated new bone formation in treated defects (Figure 6L and Figure S8J). Histological features were similar between P5 and P25 groups, indicating maintenance of regenerative capacity after prolonged culture. CD31 immunofluorescence on calvarial defect sections at 8 weeks also revealed abundant endothelial structures in organoid‐treated defects (Figure 6M), and at 4 weeks we detected early vascular infiltration (Figure S8K).

Together, these findings demonstrate that engineered periosteal organoids promote organized bone regeneration in both long‐bone and calvarial defects. The comparable regenerative performance between early‐ and late‐passage organoids indicates that extended in vitro expansion does not compromise in vivo repair ability, supporting the concept that engineered periosteal organoids provide a stable pro‐regenerative niche capable of functioning across distinct skeletal sites.

## 3 Discussion

In this study, we establish a bona fide periosteum organoid system that meets core organoid criteria: long‐term expandability, native cellular heterogeneity, and functional competence. Unlike previous models that rely on preselected progenitors or limited‐passaging cultures [21, 22, 23, 24, 25], our organoids are directly derived from enzymatically digested periosteum and maintain stable growth for over 20 passages without loss of morphology, proliferative capacity, or genomic stability, surpassing existing systems. This stability not only ensures experimental reproducibility but also provides a versatile platform for systematic mechanistic studies and in vivo functional assays, effectively overcoming a major technical bottleneck in periosteal research. Under optimized culture conditions, these periosteum organoids form rapidly within 3–5 days and remain viable, exhibiting active remodeling for more than 28 days. Collectively, these characteristics establish our periosteum organoid system as a robust and scalable platform for investigating skeletal biology and advancing regenerative therapies.

Bone regeneration poses a unique biological challenge, as effective repair requires reconstruction of a multifunctional tissue rather than simple replacement of mineralized matrix. Successful regeneration depends on coordinated integration of osteogenic, chondrogenic, vascular, neural, and hematopoietic components [44, 45]. Recent advances in bone organoid technologies, including hydrogel‐based systems, bone‐mimetic scaffolds, and engineered biomaterials, have enabled rapid formation of bone‐like structures and partial defect repair [22, 46, 47, 48, 49]. However, these approaches primarily emphasize the structural output of regeneration and often rely on material‐driven cues to induce mineralization. Although material properties such as matrix stiffness or composite scaffolds can modulate osteogenesis and enhance mineral deposition [50], such strategies do not inherently preserve the endogenous regenerative hierarchies that govern bone repair in vivo.

Within this context, our periosteum‐derived organoids offer a conceptually distinct strategy. Rather than focusing on engineered bone‐mimetic architectures or material‐driven mineralization, our periosteum‐derived organoids aim to preserve the native cellular composition and niche topology of adult periosteum (Figure 2). The periosteum is a key in vivo source of skeletal stem and progenitor cells that act in both acute regeneration and long‐term homeostasis. Single‐cell transcriptomic analyses indicate that periosteal progenitors are primary contributors to osteochondral repair following fracture; in some injury models, adjacent muscle‐derived progenitors also participate [51]. Importantly, cells from the fibrous layer of the periosteum can form neo‐skeletal stem cells (neo‐SSCs) that underpin prolonged maintenance of bone tissue [15]. By maintaining this endogenous regenerative niche ex vivo, periosteum organoids shift the focus of bone regeneration from constructing mineralized tissue to reconstructing the biological system that produces bone.

To determine whether the regenerative benefit depends on the intact 3D architecture of the organoids, we compared the outcomes of implanting equal numbers of cells delivered either as intact organoids or as dissociated single cells re‐embedded in Matrigel. At 2 weeks post‐implantation, dissociated single cells generated cartilage with no difference in tissue morphology compared to intact organoids. Notably, freshly isolated periosteal cells—which are transplanted as single cells without any pre‐existing topology—also formed abundant bone tissue at 8 weeks (Figure 3B,C). These observations indicate that the core driver of bone regeneration is the preservation of functional osteogenic cell populations rather than the intact 3D topology of the organoids.

Despite the direct contribution of periosteal organoids in providing functional osteogenic cell populations to form ectopic bone in nude mice, we must note a limitation in tracking cell fate in the rat defect models. Because both the host and the graft were of rat origin, we could not use species‐specific markers (e.g., RT1a) to distinguish donor‐derived cells from host cells. However, in the critical‐sized calvarial defect model, defects implanted with Matrigel alone failed to heal, whereas organoid‐treated defects were filled with substantial newly formed bone. Together with our subcutaneous transplantation experiments showing that newly formed bone and cartilage were derived from organoids (Figure 3H–J), these results strongly suggest that the newly formed bone and cartilage in the defect models also originate from the transplanted organoids. Our findings therefore support a model in which effective skeletal repair is driven not by engineered mineralization per se, but by preservation of the periosteal regenerative hierarchy that orchestrates coordinated osteogenesis, chondrogenesis, and niche remodeling in vivo.

We also examined whether the marrow‐like structures within the grafts represented genuine hematopoietic microenvironments rather than inflammatory infiltrates. Flow cytometry revealed that immune cells (neutrophils and macrophages) infiltrated transiently and resolved by 4 weeks, whereas hematopoietic progenitors (KSL, MEP, GMP, CMP) persisted at 8 weeks. Morphologically, the graft‐derived marrow cavities closely resembled native bone marrow, containing erythroid, granulocytic, and lymphoid precursors, megakaryocytes, osteoblasts, and sinusoids. Together, these data support the formation of a hematopoietic‐supportive niche within the grafts, although we acknowledge that definitive demonstration of functional HSC niche activity (e.g., long‐term reconstitution assays) was not performed in this study.

Beyond their osteogenic capacity, the periosteum organoids we established also provide a supportive ex vivo niche for HSCs. Long‐term maintenance of HSCs in vitro remains a major challenge, as conventional culture conditions often drive HSCs out of quiescence, leading to metabolic stress, oxidative damage, and progressive functional decline [52, 53]. In contrast, direct co‐culture with periosteum organoids preserves primitive LSK/CD150^+^ phenotypes while markedly reducing senescence markers, intracellular ROS, and metabolic dysregulation. These convergent readouts‐together with more homogeneous TMRE distributions and reduced β‐gal activity‐indicate that organoids recreate a slow‐cycling, maintenance‐promoting microenvironment that favors slow‐cycling state rather than activation; this interpretation is consistent with prior work showing that elevated ROS limits HSC self‐renewal and repopulating capacity [54]. The injury‐responsive niche state modeled by periosteum organoids differs from conventional bone‐marrow‐centered organoids, which predominantly recapitulate mature, homeostatic niches [54, 55, 56]. Existing studies show that precise microenvironmental engineering‐either via optimized factor cocktails to expand functional HSCs ex vivo [53] or via tuning matrix stiffness to rejuvenate HSCs [57] ‐can preserve HSC competence. Consistent with these reports, our organoids act as a composite niche that preserves native cellular constituents, extracellular matrix, and niche‐derived biochemical cues to maintain HSC in a slow‐cycling state and delay ex vivo functional aging. However, when we directly tested long‐term repopulating capacity via competitive transplantation, HSCs co‐cultured with periosteal organoids for 14 days failed to achieve detectable multilineage reconstitution in vivo. Thus, while our organoid system effectively delays HSC functional decline, reduces oxidative stress and senescence, and maintains a primitive phenotype ex vivo, it does not preserve durable repopulating capacity under the current culture conditions. Future work should perform in vivo reconstitution experiments and identify necessary organoid‐derived cues through secretion profiling and perturbation.

At the molecular level, we identified the transcription factor ZFP575 as being selectively retained in 3D organoids and required for in vivo osteogenic competence: its knockdown does not disrupt organoid morphology in vitro but markedly impairs bone formation after transplantation, indicating ZFP575 as a tractable entry point for understanding how 3D niche cues maintain lineage competence. This uncoupling between in vitro appearance and in vivo function is a recognized feature of skeletal progenitor biology and underscores the necessity of functional transplantation assays to define true osteogenic potential [58, 59]. Defining ZFP575's downstream targets should help elucidate the mechanistic links among 3D architecture, matrix/metabolic state, and differentiation potential. Consistent with this, single‐cell composition dynamics across passages show early depletion of non‐mesenchymal lineages (endothelial, immune, B cells) while a core mesenchymal compartment (mesenchymal progenitors, osteoprogenitors, ECM‐producing fibroblasts) is robustly preserved. The flow cytometric validation of B cells and macrophages in freshly isolated periosteal cells and their rapid depletion upon passaging (Figure S2C,D) corroborates our scRNA‐seq findings and further supports the conclusion that non‐mesenchymal lineages are progressively lost under culture conditions optimized for periosteal mesenchymal cell maintenance. This dichotomy explains the functional phenotypes: the retained mesenchymal core supports sustained osteogenesis and partial HSC maintenance, whereas loss of vascular and immune elements limits the model's ability to recapitulate the full bone‐marrow niche [60, 61] and suggests that ZFP575 may act by maintaining or regulating these mesenchymal subpopulations and their micro‐environment.

## 4 Conclusion

The strengths of our system‐adult tissue origin, long‐term stability, and dual functionality (osteogenic and hematopoietic support)‐make it a versatile tool for both basic and preclinical studies. By coupling mechanistic dissection with efforts to reconstitute vascular and immune elements and to humanize the platform, periosteum organoids can accelerate translation of regeneration‐focused therapies and provide a tractable model for studying skeletal aging and niche dysfunction.

## 5 Experimental Section

### 5.1 Reagents and Antibodies

The following antibodies were purchased from Santa Cruz Biotechnology (Santa Cruz, CA, USA): Santa mouse anti‐goat IgG‐CFL 555 (1:500, sc‐516242), m‐IgGκ BP‐CFL 647 (1:500, sc‐516179), mouse anti‐rabbit IgG‐FITC (1:500, sc‐2359). Endomucin (1:50, sc‐65495). Desmin (1:100, CL488‐16520) was from Proteintech Group, Inc. (Wuhan, China). For flow cytometry, the following were purchased from Biolegend. (San Diego, CA, USA): APC anti‐mouse CD2 (1:200, 100112), APC anti‐mouse CD5 (1:200, 100626), APC anti‐mouse CD8a (1:200, 00712), PE/Cyanine7 anti‐mouse CD150 (1:200, 115914), FITC anti‐mouse CD48 (1:200, 103404), RT1a (1:100, 205201) and CD45 (1:200, 17‐0459‐42). APC Anti‐Mouse Ly‐6G (1:200, 20–5931), APC Anti‐Mouse TER‐119 (1:200, 20–5921), and APC Anti‐Human/Mouse CD45R (1:200, 20–0452) were from Tonbo Biosciences (Auburn, CA, USA). The following antibodies were purchased from Abcam (Cambridge, MA, USA)): OPN (1:100, ab11503), Periostin (1:50, ab315104) and COL2 (1:50, ab34712) for IF. COL10 (1:50, 14‐9771‐80) and SDF1 (1:100, PA5‐114344) were from Thermo Fisher Scientific (Waltham, MA, USA). ZFP575 (1:1000, bs‐13588R) was from Bioss (Beijing, China).

### 5.2 Animals

C57BL/6J mice, Balb/C‐nude mice, and Sprague‐Dawley (SD) rats were obtained from the Department of Laboratory Animal Science, Peking University Health Science Center. 8‐12‐week‐old male C57BL/6J mice were used for bone marrow collection. Balb/C‐nude mice aged 8 weeks were used for subcutaneous heterotopic transplantation experiments. Periosteal tissue was collected from 6‐week‐old male SD rats. Critical‐sized bone defect models were established in 12‐week‐old male SD rats. All animals were housed in a specific pathogen‐free (SPF) barrier facility under controlled conditions (22°C, 50% humidity, 12‐h light/dark cycle). Animals were randomly assigned to experimental groups. Investigators were not blinded during experimentation or outcome assessment. No sex‐specific analyses were performed unless otherwise stated. All animal experiments were approved by the Peking University Animal Ethics Committee (Approval No. BDKQ‐202504120540) and conducted in accordance with ARRIVE guidelines.

### 5.3 Primary Periosteal Cell Isolation

SD rats were selected for this study because the periosteum is an extremely thin tissue composed of only a few cell layers; thus, larger animals provide a greater tissue yield, which is essential for establishing a stable and expandable periosteal organoid bank. Rats were euthanized by cervical dislocation and disinfected in 75% ethanol. Bilateral femurs and tibias were dissected, muscles were removed, and bones were placed in pre‐cooled isolation buffer (PBS supplemented with 4% (v/v) FBS and 2% (v/v) penicillin‐streptomycin). Periosteum was gently scraped off using a periosteal elevator on ice and collected in buffer. Tissue was transferred to a 1.5 mL tube, minced finely with sterile scissors in 0.5 mL collagenase digestion solution, and vortexed. After adjusting the volume to 0.5–0.8 mL, digestion proceeded in a 37°C shaker (800 rpm) for 30 min. The digest was filtered through a 70 µm strainer, neutralized with an equal volume of buffer, and centrifuged (1000 rpm, 5 min, 4°C). A second digestion was performed to maximize yield. The cell pellet was briefly resuspended in < 1 mL sterile water for 5–6 s to lyse red blood cells, followed immediately by adding 3 volumes of isolation buffer. After centrifugation, cells were resuspended in 400–800 µL isolation buffer, stained with flow cytometry antibodies for 30 min at 4°C, washed, filtered, and sorted.

### 5.4 Periosteal Organoid Culture

Sorted periosteal cells were resuspended in growth factor‐reduced Matrigel (Corning, 356231). 40 µL drops were plated in pre‐warmed 12‐well plates and polymerized at 37°C for ≥ 15 min. After polymerization, 1 mL of organoid growth medium was added per well. The medium consisted of MEM α (Gibco, C12571500CP), 2% penicillin/streptomycin (Gibco, 15140122), 10 mm HEPES (Gibco,15630080), 2 mm GlutaMAX (Thermofish, 35050061), 1× B‐27 (Gibco, 17504044), 1× N2 supplements (Gibco, 17502048), 20 ng/mL bFGF (PeproTech, 100‐18B‐50UG), 20 ng/mL OSM (PeproTech, 300–10H‐10UG), 20 ng/mL EGF (PeproTech, AF‐100‐15‐100UG), 40 ng/mL IGF (PeproTech, 100‐11‐1MG), and 15% chicken embryo extract (MP, 092850145). 20 µm ROCK inhibitor Y27632 (STEMCELL Technologies, 72304) was added for the first 2–3 days to inhibit anoikis. Cultures were maintained at 37°C, 5% CO_2_, with medium changes every 2–3 days for the first 7–14 days. Organoids were passaged upon confluence.

### 5.5 Organoid Passaging

Medium was removed, and organoids were gently washed with PBS. Matrigel domes were detached using a pipette, and the PBS‐gel mixture was transferred to a 15 mL tube. Cell Recovery Solution (Corning, 354253, 1 mL per 5 domes) was added, and digestion proceeded at 4°C for 15 min. After adding 3 volumes of organoid growth medium, the suspension was triturated 50 times with a pipette and centrifuged (250g, 5 min, 4°C). The pellet was resuspended in a 3:1 mix of Matrigel to organoid growth medium, and 40 µL drops were plated. For passaging, 2–3 drops were plated per well in 1 mL organoid medium containing 20 µm Y27632, with medium changed every other day. Passaging is effective only within the first 2–5 days of culture; day‐14 organoids are too compact to be effectively dissociated without compromising cell viability and function.

### 5.6 Organoid Cryopreservation

Organoids were passaged at least three times before cryopreservation. Mature organoid‐containing Matrigel domes were detached with cold PBS, digested with Cell Recovery Solution at 4°C for 15 min, and centrifuged (250g, 5 min). The pellet was resuspended in Freezing Medium (Procell system, PB180438), aliquoted (1 mL per cryovial), and placed in a controlled‐rate freezing container at −80°C overnight before transfer to liquid nitrogen. Typically, organoids from two domes were pooled per vial.

### 5.7 Organoid Thawing

Cryovials were retrieved from liquid nitrogen and rapidly thawed in a 37°C water bath for ∼ 2 min. Contents were transferred to 6 mL pre‐warmed organoid growth medium and centrifuged (250g, 5 min, 4°C). The pellet was resuspended in 5 mL medium, washed once, and finally resuspended in a 3:1 Matrigel‐to‐medium mix for plating as 40 µL drops. Thawed organoids were passaged at least once before use.

### 5.8 Lentiviral Transduction of Organoids

Organoids were washed with PBS, digested with Cell Recovery Solution at 4°C for 15 min, gently dissociated, centrifuged, and counted. The suspension was plated in 48‐well plates, mixed with lentivirus at the desired multiplicity of infection, and centrifuged (600g, 32°C, 1 h) to enhance infection, followed by incubation at 37°C for 6 h. The lentiviral sequences are provided in Table 1. Transduced organoids were re‐embedded in Matrigel and cultured with 20 µm Y27632 for 2–3 days. Puromycin (5 µg/mL) selection began on day 2 and lasted for 14 days, with regular monitoring of fluorescence and morphology.

**TABLE 1:** *Zfp575* knockdown lentiviral sequences.

Name	Sequences
NC	TTCTCCGAACGTGTCACGTAA
*Zfp575*sh‐1	GGTCTCAGAACCTAATCCCACTTCT
*Zfp575*sh‐2	CCGATTGTCCCAAGGCCTTCTCATA
*Zfp575*sh‐3	CAAAGCGTTCTCGTTTCCCTCCAAA

### 5.9 HSC Isolation

Long bones from euthanized mice were cleaned of muscle and placed in cold buffer. Bones were gently tapped in a mortar on ice to release marrow, avoiding grinding. The cell suspension was flushed with PBS, filtered through a 100 µm strainer, centrifuged, resuspended, and counted. Cells were stained with antibodies for lineage‐negative (Lin^−^) markers and stem cell surface markers (c‐Kit^+^Sca‐1^+^CD48^−^CD150^+^) at recommended concentrations. After staining, washing, and filtering, HSCs were isolated by fluorescence‐activated cell sorting (FACS).

### 5.10 HSC Culture

Sorted mouse HSCs were centrifuged and plated at 35 cells per well in fibronectin‐coated 48‐well plates with 400 µL pre‐warmed medium (HSC medium or co‐culture system). The standard HSC medium was StemSpan SFEM II (STEMCELL Technologies, 09605), supplemented with 1% penicillin/streptomycin, 50 ng/mL TPO (PeproTech, 315‐14‐10UG), 50 ng/mL Flt3L(PeproTech, 250‐03‐10UG), and 100 ng/mL SCF(PeproTech, 250‐31L‐10UG) [57]. Co‐culture medium was prepared by mixing standard HSC medium and periosteal organoid culture medium at a 1:1 ratio. For the co‐culture system, HSCs were cultured with periosteal organoids in co‐culture medium in suspension (without Matrigel embedding). Medium was not changed for the first 4 days, then replaced every 2 days.

### 5.11 Subcutaneous Ossification in Nude Mice

Mature periosteal organoids were harvested intact. Nude mice were anesthetized by Isoflurane. After dorsal skin disinfection, a ∼ 1 cm midline incision was made. Subcutaneous pockets were created by blunt dissection. Organoids were implanted into the pockets, and the incision was closed in layers with 5‐0 absorbable sutures. Matrigel alone served as a control. After 8 weeks, animals were euthanized, and the implantation sites were harvested, fixed in 4% paraformaldehyde, and scanned by micro‐CT (Skyscan 1276, Bruker, Belgium). Samples were then decalcified, paraffin‐embedded, and sectioned for H&E, Masson's trichrome, and IF staining.

### 5.12 Rat Critical‐Sized Femoral and Calvarial Defect Models

For the femoral defect model, rats were anesthetized with Isoflurane. The right hindlimb was shaved and disinfected. A longitudinal incision was made on the medial side of the knee joint. Muscles were separated to expose the femoral metaphysis. Under continuous saline irrigation, a 4 mm diameter full‐thickness cortical bone defect was created approximately 1 cm above the medial femoral condyle using a dental bur (1000 rpm). After irrigation to remove debris, a single organoid or an equal volume of Matrigel (control) was implanted into each defect. Muscle and skin were sutured in layers. Penicillin was administered for 3 days postoperatively. For the calvarial defect model, after anesthesia and scalp shaving/disinfection, a 1.5–2 cm sagittal skin incision was made. The periosteum over bilateral parietal bones was thoroughly elevated. Under saline cooling, two symmetrical 5 mm diameter full‐thickness bone defects were created in the parietal bones. The defect received organoids or Matrigel as an internal control. The periosteum and skin were sutured in layers with postoperative care as above. Animals were euthanized at 4 and 8 weeks post‐surgery. Femurs and calvariae were harvested, fixed in 4% PFA, and scanned by high‐resolution micro‐CT for quantitative analysis of BV and BMD. Samples were then decalcified, paraffin‐embedded, and sectioned for histological staining (H&E, Masson's trichrome, Safranin O/Fast Green) and immunofluorescence (OPN and CD31).

### 5.13 qRT‐PCR

Total RNA was extracted using Trizol reagent (Invitrogen). cDNA was synthesized with a reverse transcription kit (Takara Bio). qRT‐PCR was performed using SYBR Master Mix (Roche) on an Applied Biosystems StepOne Plus system (ABI 7900). Gene expression was analyzed using specific primer sequences for β‐actin and target genes (Tables 2 and 3).

**TABLE 2:** The primer sets for rat qPCR.

Gene(rat)	Primers (Forward)	Primers (Reverse)
*Zfp575*	5′‐ CAAGATGCTGGGTGGAAGCG ‐3′	5′‐ CAGGTCCTGGTGGGAGACTTTG ‐3′
*Tcf7*	5′‐ AGGAGGCGAAGAAGCCAATCA ‐3′	5′‐ GATAATGCATGCCACCTGCGAC ‐3′
*Sox6*	5′‐ GAAAGATTTCCATGGCTTTGTCACT ‐3′	5′‐ TACACGAGGATCACCCCAGC ‐3′
*Rarg*	5′‐ CATTACCGCGAGTCACTAACACC ‐3′	5′‐ GGACCTGGTAGAAACTGCTGGA ‐3′
*Tsc22d1*	5′‐ AGGTGCTACCGCTGACAACA ‐3′	5′‐ AATGGCTTTTCACCAGATCCATAGC ‐3′
*β‐actin*	5′‐ CCGCGAGTACAACCTTCTTGC ‐3′	5′‐ TCATCCATGGCGAACTGGTGG ‐3′

**TABLE 3:** The primer sets for mouse qPCR.

Gene(mouse)	Primers (Forward)	Primers (Reverse)
*Gapdh*	5′‐ ACAGCAACTCCCACTCTTCCAC‐3′	5′‐ AGTTGGGATAGGGCCTCTCTTG‐3′
*p16Ink4a*	5′‐ GAACTCTTTCGGTCGTACCC ‐3′	5′‐ CGAATCTGCACCGTAGTTGA ‐3′
*p19Arf*	5′‐ GGGTTTTCTTGGTGAAGTTCG ‐3′	5′‐ TTGCCCATCATCATCACCT ‐3′
*Tnfα*	5′‐ CCCACGTCGTAGCAAACCAC ‐3′	5′‐ GTACAACCCATCGGCTGGCA ‐3′
*Trp53*	5′‐ CAGTCTACTTCCCGCCATAA ‐3′	5′‐ GTCTCAGCCCTGAAGTCATAAG ‐3′
*Ki67*	5′‐ CCATCATTGACCGCTCCTTTAGGTA ‐3′	5′‐ TTGACCTTCCCCATCAGGGT ‐3′

### 5.14 Western Blot Analysis

Rat periosteal organoids were lysed using RIPA lysis buffer (Solarbio, R0010). The samples were then subjected to ultrasound homogenization on ice for protein extraction. Protein concentrations were determined using a BCA protein assay kit (Thermos Scientific, 23227). Subsequently, the proteins were separated by electrophoresis on a 10% SDS‐PAGE gel for 1 h. Electrophoresis was stopped when the bromophenol blue dye front migrated to the bottom edge of the gel. Proteins were transferred onto activated PVDF membranes under constant current conditions at 400 mA. The membranes were then blocked with TBST containing 5% non‐fat milk for 2 h. This was followed by overnight incubation with primary antibodies: anti‐GAPDH (1:5000, Proteintech, 10494‐1‐AP) and ZFP575 (1:1000, Bioss, bs‐13588R). After three washes with TBST, the membranes were incubated with HRP‐conjugated secondary antibody (1:10000, Proteintech, SA00001‐2) for 1 h at room temperature. Following thorough washing with TBST, signals were visualized using an ECL chemiluminescence reagent and analyzed using ImageJ software (v1.8.0, NIH).

### 5.15 Immunofluorescence and H&E Staining

Samples were fixed in 4% paraformaldehyde for 2 days, dehydrated through graded ethanol, embedded in paraffin, and sectioned. For H&E staining, sections were stained with hematoxylin and eosin. For immunofluorescence, after antigen retrieval in citrate buffer, sections were incubated with primary antibodies overnight at 4°C, followed by fluorescent secondary antibodies for 1 h at room temperature. Nuclei were counterstained with DAPI, and sections were mounted. Images were captured using a fluorescence microscope (Olympus FV3000).

### 5.16 Statistics and Reproducibility

Statistical analyses were performed using GraphPad Prism 10. Data are presented as mean ± standard deviation. Student's *t*‐test was used for comparisons between two groups. One‐way ANOVA was used for comparisons among multiple groups. A *P*‐value < 0.05 was considered statistically significant. The number of independent experiments and replicates (n) is indicated in the figure legends. All experiments were performed with at least three biologically independent replicates, yielding consistent results. Animals were randomly assigned to experimental groups. Investigators were not blinded during experimentation or outcome assessment. No sex‐specific analyses were performed unless otherwise stated. All experimental units that successfully completed the predefined experimental protocol without protocol deviations were included. Animal death prior to endpoint and obvious technical errors were excluded.

### 5.17 Single Cell Library Preparation and Sequencing

Single‐cell suspensions were processed with the 10x Genomics Chromium Single Cell 3’ v3 chemistry to generate barcoded scRNA‐seq libraries. Libraries were sequenced as paired‐end reads on an Illumina NovaSeq platform (PE150) targeting ∼ 100 000 reads per cell.

### 5.18 Preprocessing and Generation of Feature Barcode Matrices

FASTQ files were provided by the sequencing facility, and read‐level QC was performed with fastp [62]. Each library was processed independently with Cell Ranger (v9.0.1) [63] for alignment, barcode/UMI processing, and gene counting against the rat reference refdata‐gex‐mRatBN7‐2‐2024‐A using default parameters, producing per‐sample filtered feature‐barcode matrices. Cell Ranger aggr was not used; samples were integrated downstream with Harmony.

### 5.19 Data Import and Metadata Annotation

Filtered feature‐barcode matrices were imported into Seurat (v5.3.1) [64] using Read10X and Create Seurat Object (min. cells = 3; min. features = 200). Sample metadata (sample ID, passage, replicate) were added to each Seurat object.

### 5.20 Quality Control and Doublet Removal

Mitochondrial transcript percentage was computed per sample using genes matching the pattern ^mt‐ (case‐insensitive). An adaptive two‐stage QC strategy was applied. First, permissive filtering was used to retain cells for robust doublet detection: sample‐specific cutoffs for nFeature_RNA, nCount_RNA, and percent.mt were derived from median absolute deviation (MAD) bounds with additional hard constraints (genes: 300–25 000; UMIs: 500‐100 000; mitochondrial % ≤ the smaller of 20 and the larger of 8 and the sample‐specific upper_bound. Doublets were identified per sample with scDblFinder (v1.22.0; seed = 123), and only singlets were retained. A stricter filtering was then applied to singlets (genes: 500–10 000; UMIs: 1000–50 000; mitochondrial % ≤ the smaller of 10 and the larger of 5 and the sample‐specific upper_bound.

### 5.21 Normalization and Variance Stabilization

Each sample was normalized with SCTransform using the glmGamPoi method (method = “glmGamPoi”), regressing out percent.mt and nCount_RNA. Variable features were set to 3000, and all genes were retained (return.only.var.genes = FALSE; seed.use = 42).

### 5.22 Dimensionality Reduction, Integration, Clustering, and Visualization

PCA was performed on SCTransform residuals and the top 30 PCs were retained. Batch correction and cross sample integration were performed with Harmony (v1.2.4)65 using sample as the batch variable and 30 PCs as input. A shared nearest neighbor (SNN) graph was constructed with Seurat FindNeighbors, followed by graph based clustering (FindClusters) at resolution = 0.4. Two dimensional visualization was generated with UMAP (RunUMAP) using default parameters.

### 5.23 Cell Type Annotation

Cluster marker genes were identified with Seurat Find All Markers. Markers were ranked by a composite score (avg_log2FC × (pct.1‐pct.2)), and clusters were annotated based on canonical marker expression.

### 5.24 Functional Enrichment Analysis (GO)

GO biological process enrichment was performed per cluster using cluster Profiler. For each cluster, significantly upregulated marker genes (adjusted *P* < 0.05 and avg_log2FC > 0) were selected, retaining up to 300 genes per cluster ranked by adjusted *P* value and log2 fold change; clusters with fewer than 10 input genes were excluded. Gene symbols were converted to Entrez IDs via org.Rn.eg.db. The background universe comprised all marker genes that could be mapped to Entrez IDs. Enrichment was tested with enrich GO and Benjamini–Hochberg correction; terms with adjusted *P* < 0.05 were considered significant. Results were visualized as dot plots (top 15 terms per cluster) and exported as a multi page vector PDF.

### 5.25 Copy Number Variation Inference

Large scale CNV patterns were inferred with infer CNV (v1.24.0). To limit memory use and balance cell counts across passages, cells were randomly down‐sampled per passage (set. seed = 123) to a maximum of 2000 cells per passage before layer joining and matrix extraction. Seurat v5 RNA layers were merged using Join Layers, and the RNA counts matrix was extracted for infer CNV. A gene order file was derived from the reference GTF and filtered to genes present in the counts matrix. Fresh cells served as the reference group; sex chromosomes and the mitochondrial chromosome were excluded. Infer CNV was run with cutoff = 0.1, denoise = TRUE, HMM = FALSE, and clustering by group (passage) using 6 threads.

### 5.26 Cell‐Cell Communication Analysis

Cell Chat (v2.2.0)66 was used to infer and compare intercellular communication between Fresh and Organoid_all. Seurat v5 RNA layers were merged with Join Layers, and counts were extracted. Ensembl IDs were mapped to rat gene symbols via biomaRt to ensure compatibility with ligand–receptor databases. Cell Chat objects were created using cell type as the grouping variable; the mouse CellChat database was used, restricting to secreted signaling interactions. Communication probabilities were computed with population. Size = TRUE, and interactions filtered with min. cells = 10. Fresh and Organoid_all Cell Chat objects were merged for comparative analyses.

## Author Contributions

Y. Z. and P. Z. conceived the study; J. Q. and X. G. performed most of the experiments, with significant input from Z. L., H. Z., W. S., H. L.; L.G. and L.F. analyzed the sequencing data with the assistance of P. Z.; Y.Z. and P. Z. contributed to data interpretation; Y. Z., P. Z., and J.Q. wrote the manuscript and revised it based on feedback from all the co‐authors.

## Funding

This work was supported by the National Natural Science Foundation of China (Nos. 82571063, 82530030, 82270954, and 82501102), the Beijing Natural Science Foundation (Nos. 7242174), the Clinical Research Foundation of Peking University School and Hospital of Stomatology (PKUSS‐2024CRF1002, CZPKUSS‐2025CRF1002, PKUSS‐2023CRF1005), the Non‐profit Central Research Institute Fund of Chinese Academy of Medical Sciences (2023‐PT320‐09), and the China Postdoctoral Science Foundation (2025M771755 and 2025M781682).

## Conflicts of Interest

The authors declare no conflicts of interest.

## Supporting information




**Supporting File 1**: advs78380‐sup‐0001‐SuppMat.docx.

**Supporting File 2**: advs78380‐sup‐0002‐SuppMat.docx.

**Supporting File 3**: advs78380‐sup‐0003‐VideoS1.mp4.

**Supporting File 4**: advs78380‐sup‐0004‐VideoS2.mp4.

## Data Availability

The datasets supporting the findings of this study have been deposited in the Genome Sequence Archive (GSA) at the National Genomics Data Center, China National Center for Bioinformation / Beijing Institute of Genomics, Chinese Academy of Sciences, under the accession numbers CRA037658, and are publicly accessible at https://ngdc.cncb.ac.cn/gsa.
