# Supplementary material for: Long‐Term Expandable Periosteal Organoids Reconstruct a Functional Regenerative Niche That Support Hematopoietic Stem Cell Maintenance and Bone Repair
Source: Adv Sci (Weinh). 2026 Oct 22:e78380. Online ahead of print. doi: 10.1002/advs.78380 (PMC13690986; doi:10.1002/advs.78380)
Supplement: Supplementary file 1 [file ADVS-9999-e78380-s002.docx]

Supplemental information

**Uncropped blot for Figure S7C**

**ZFP575 for replicate 1:**

70kD

50kD

40kD

35kD

25kD

**GAPDH for replicate 1:**

70kD

50kD

20kD

35kD

40kD

Photograph of uncropped membrane for replicate 1:

**ZFP575 for replicate 2:**

50kD

70kD

**GAPDH for replicate 2:**

20kD

40kD

35kD

The two cut membrane strips for replicate 2 were placed side by side and photographed together.

**ZFP575 for replicate 3:**

50kD

70kD

**GAPDH for replicate 3:**

20kD

40kD

35kD

The two cut membrane strips for replicate 3 were placed side by side and photographed together.
