# Supplementary material for: Long‐Term Expandable Periosteal Organoids Reconstruct a Functional Regenerative Niche That Support Hematopoietic Stem Cell Maintenance and Bone Repair
Source: Adv Sci (Weinh). 2026 Oct 22:e78380. Online ahead of print. doi: 10.1002/advs.78380 (PMC13690986; doi:10.1002/advs.78380)
Supplement: Supplementary file 2 [file ADVS-9999-e78380-s003.docx]

Supporting Information

Long-term expandable periosteal organoids reconstruct a functional regenerative niche that support hematopoietic stem cell maintenance and bone repair

Jia Qing#, Lanxin Gu#, Lipeng Fu, Xin Gao, Zechuan Li, Hui Zhang, Wenpeng Song, Huixin Lv, Yongsheng Zhou*, Ping Zhang*

**Figure S1. Supplementary characterization of periosteal organoid culture and isolation.**

(A) Dissection image showing periosteal tissue isolated from rat femurs. (B) Representative FACS gating strategy for sorting periosteal cells. (C) Growth curves (organoid diameter versus culture time) for organoids derived from three independent rat batches over 14 days. Each point represents mean ± SD (n=3 organoids per time point). (D) Organoid diameter reduction ratio during organoid maturation across the three rat batches. Data are presented as mean ± SEM (n=3). P-values were calculated using one-way ANOVA with Tukey's multiple comparisons test. ns, not significant. (E) Live/Dead staining of periosteal organoids at days 7, 14, 21, and 28 (Calcein-AM, green, laser 488; Ethidium homodimer-1, red, laser 647). Scale bar: 1300 μm. (F) Quantification of live cell percentage in organoids over time. Data are presented as mean ± SEM (n=6). P-values were calculated using one-way ANOVA with Tukey's multiple comparisons test. ns, not significant. (G) Diameter reduction ratio during organoid maturation across passages P0 to P20. Data are presented as mean ± SEM (n≥3). P-values were calculated using one-way ANOVA with Tukey's multiple comparisons test. ns, not significant. (H) Growth dynamics of P5 versus P20 organoids assessed by phase exposure time. Data are mean ± SD; n = 3. (I) Immunofluorescence staining for fibronectin (green) in periosteal organoid and freshly isolated rat periosteal tissue; nuclei stained with DAPI (blue). Scale bar: 60 μm. (J) Immunofluorescence staining for connexin 43 (red) in periosteal organoid and freshly isolated rat periosteal tissue; nuclei stained with DAPI (blue). Scale bar: 60 μm.

**Figure S2. Dynamic changes in cellular composition of periosteal organoids during serial passaging.**

(A) UMAPs of single cells from fresh periosteum and organoids at indicated passages (Fresh, P0–P6, P20), plotted separately to show sample‑specific distribution. (B) Passage‑resolved longitudinal profiles showing changes in cell‑type proportions across serial passaging (P0–P20). Data points are mean ± SD; exact n per passage is provided in Methods. (C) Representative flow cytometry plots showing B cell (top) and macrophage (bottom) populations in freshly isolated periosteal cells, passage 0 organoids, and passage 1 organoids. (D) Quantification of B cells and macrophages in freshly isolated periosteal cells, passage 0 organoids, and passage 1 organoids by flow cytometry. Data are presented as mean ± SEM (n=3). Statistical significance was determined by one‑way ANOVA followed by Tukey’s post‑hoc test; ns, not significant, *p < 0.05, ***p < 0.01, ****P < 0.0001 versus control.

**Figure S3. Functional enrichment, signaling network analysis, and CNV stability of periosteal organoids.**

(A) GO biological process enrichment for cluster‑defining marker genes. Dot plots show Gene Ratio (x‑axis), adjusted *P* value (color), and gene count (dot size); only the top enriched terms per cluster are shown. (B) Comparison of intercellular communication strength (information flow) for selected signaling pathways between fresh periosteum and pooled organoids, inferred by Cell Chat. (C) InferCNV output summarizing large‑scale copy‑number variation across passages; fresh cells (reference) are shown at top and organoid observations below (genomic position on x‑axis; CNV signal on y‑axis).

**Figure S4. Supplementary histological analysis of periosteal organoid grafts at 8 weeks after subcutaneous transplantation.**

(A) Quantification of bone volume (BV), bone volume fraction (BV/TV), and bone mineral density (BMD) from micro-CT analysis of 8-week grafts. Data are mean ± SEM; n = 3. (B) Safranin O/Fast Green staining of 8-week grafts derived from fresh periosteum and organoids. Scale bar: 80 μm. (C) Masson's trichrome staining of 8-week grafts derived from fresh periosteum and organoids. Scale bar: 80 μm. (D) H&E staining of 8-week grafts from cryopreserved organoids at P26. Scale bars: 500μm, 50μm. (E) Oil Red O staining of 8-week grafts, with adjacent adipose tissue as a positive control. Scale bar: 50 μm.

**Figure S5. Time-course analysis of periosteal organoid grafts after subcutaneous transplantation.**

(A) Representative 3D micro-CT reconstructions of organoid grafts harvested at 1, 2, 4, and 8 weeks post-transplantation and longitudinal quantification of BV and BMD in organoid grafts over time. Data are presented as mean ± SEM (n=3). Statistical significance was determined by one‑way ANOVA followed by Tukey’s post‑hoc test; ns, not significant, *p < 0.05, ***p < 0.01, ****P < 0.0001 versus control. (B-F) Representative images of 1-week organoid grafts: gross view (B), Safranin O/Fast Green (C), H&E (D-E), and immunofluorescence for COL2 (green, laser 488) and COL10 (red, laser 647) (F). Scale bars: 400μm, 200μm, 40μm and 50 μm. (G-K) Representative images of 2-week grafts: gross view (G), Safranin O/Fast Green staining (H), H&E (I-J), and immunofluorescence for COL2 (green) and COL10 (red) (K). Scale bars: 400μm, 40μm and 50 μm. (L-P) Representative images of 4-week organoid grafts: gross view (L), Safranin O/Fast Green staining (M), H&E (N-O), and immunofluorescence for COL2 (green, laser 488) and COL10 (red, laser 647) (P). Scale bars: 400μm, 40μm and 50 μm. (Q-T) Representative images of 8-week grafts: gross view (Q), Safranin O/Fast Green staining (R), and H&E (S-T). Scale bars: 400μm and 40μm. (U) Flow cytometry analysis of hematopoietic progenitor subsets (GMP, CMP, MEP) recovered from 8-week organoid grafts. (V) Representative flow cytometric gating plots showing neutrophils (top) and macrophages (bottom) in grafts at indicated time points post-transplantation. (W) Quantification by flow cytometry of neutrophils (top) and macrophages in grafts at indicated time points post-transplantation. Data are presented as mean ± SD (n=3). (X) Representative H&E staining images of normal rat femoral bone marrow (left) and marrow‑like structures within periosteal organoid grafts (right). Black arrows indicate megakaryocytes; green arrows indicate sinusoids containing mature erythrocytes; brown arrows indicate proerythroblasts; yellow arrows indicate segmented neutrophils; grey gradient arrows indicate lymphocytes; orange arrows indicate osteoblasts lining the bone surface. Scale bars: 400 μm and 40 μm.

**Figure S6. Supplementary data related to HSC co-culture with periosteal organoids.**

(A) Representative FACS gating strategy for isolation of mouse hematopoietic stem cells (HSCs: Lin⁻c-Kit⁺Sca-1⁺CD48⁻CD150⁺). Sequential gates shown: singlets, live cells, Lin^−^, c‑Kit^+^, Sca‑1^+^ population and final CD48^−^ CD150^+^ HSC gate. (B) Violin plots of selected HSC‑supportive factor expression in fresh periosteum and organoids at indicated passages (Fresh, P0-P6, P20) from scRNA‑seq. (C) Flow cytometry quantification of cell cycle distribution in Lin⁻ cells harvested from HSC cultures after 10 days. Data are presented as mean ± SEM (n=3). *P*-values were calculated using one-way ANOVA with Tukey's multiple comparisons test. **P* < 0.05, *****P* < 0.0001. (D) Flow cytometry quantification of peripheral blood chimerism at 4 weeks post‑transplantation. Data are presented as mean ± SEM (n=5). *P*-values were calculated using one-way ANOVA with Tukey's multiple comparisons test. ns, not significant.

**Figure S7. Supplementary characterization of ZFP575 function in periosteal organoids.**

(A) Quantitative RT-PCR analysis of *Tcf7*, *Tsc22d1*, *Rarg* and *Sox6* expression in fresh periosteum, 2D-cultured cells, and organoids. Data are presented as mean ± SEM (n=3). *P*-values were calculated using one-way ANOVA with Tukey's multiple comparisons test. ns, not significant, ***P* < 0.01, ****P* < 0.001, *****P* < 0.0001. (B) Single-cell RNA sequencing (scRNA-seq) analysis showing transcription factor expression across distinct cell populations. (C to E) RNA and protein expression of ZFP575 in organoids after 14 days of puromycin selection. GAPDH served as loading control. Data are presented as mean ± SEM (n=3). *P*-values were calculated using one-way ANOVA with Tukey's multiple comparisons test. ns, not significant, ***P* < 0.01, *****P* < 0.0001. (F) Organoid diameters at day 14 in control (NC) and *Zfp575*-knockdown groups. Data are presented as mean ± SEM (n≥3). *P*-values were calculated using one-way ANOVA with Tukey's multiple comparisons test. ns, not significant. (G) Quantitative analysis of organoid diameter reduction ratio during culture. Data are presented as mean ± SEM (n≥3). *P*-values were calculated using one-way ANOVA with Tukey's multiple comparisons test. ns, not significant. (H) Time course of GFP-positive cell proportion in NC, *Zfp575*sh-2 and *Zfp575*sh-3 organoids during puromycin selection. (I and J) Masson's trichrome and Safranin O/Fast Green staining of 8-week grafts from NC and *Zfp575*-knockdown organoids. Scale bars: 400 μm.

**Figure S8. Supplementary analysis of periosteal organoid-mediated bone regeneration.**

(A and B) Masson's trichrome (A) and Safranin O/Fast Green (B) staining of femoral defect areas at 8 weeks. Scale bar: 200 μm. (C) Representative micro-CT reconstruction of rat calvarial defects 4 weeks after transplantation with Matrigel, P5 organoids, or P25 organoids. Scale bar: 5 mm. (D to F) Quantitative analysis of BMD (D), BV/TV (E), and BV (F) in calvarial defects at 4 weeks. Data are presented as mean ± SEM (n=3 biological replicates). *P*-values were calculated using one-way ANOVA with Tukey's multiple comparisons test. ns, not significant, **P* < 0.05, **P < 0.001. (G) H&E staining of calvarial defect areas at 4 weeks. Scale bars: 2000 μm (top), 400 μm (bottom). (H) Masson's trichrome staining of calvarial defect areas at 4 weeks (top) and 8 weeks (bottom). Scale bar: 2000 μm. (I) Safranin O/Fast Green staining of calvarial defect areas at 4 weeks (top) and 8 weeks (bottom). Scale bar: 2000 μm.(J) Immunofluorescence staining for OPN (purple, laser 555) of calvarial defect areas at 4 weeks. White dashed lines indicate the newly formed bone region. Scale bar: 200 μm. (K) Immunofluorescence staining for CD31 (green, laser 488) in calvarial defect areas at 4 weeks. White dashed lines indicate the newly formed bone region and white arrows indicate CD31^+^ endothelial structures. Scale bar: 200 μm.
